# The structure of intact and active Photosystem II from *Arabidopsis thaliana* at 2.44 Å resolution

**DOI:** 10.1111/nph.71085

**Published:** 2026-04-10

**Authors:** Jack Forsman, André T. Graça, Abuzer Orkun Aydin, Michael Hall, Rana Hussein, Wolfgang P. Schröder, Johannes Messinger

**Affiliations:** ^1^ Department of Chemistry Umeå University Umeå SE 90187 Sweden; ^2^ Molecular Biomimetics, Department of Chemistry‐Ångström Uppsala University Uppsala SE 75120 Sweden; ^3^ European Molecular Biology Laboratory EMBL Grenoble Grenoble 38042 France; ^4^ Umeå Plant Science Center, Department of Plant Physiology Umeå University Umeå SE 90187 Sweden; ^5^ Department of Biology Humboldt‐Universität zu Berlin Berlin D 10099 Germany; ^6^ Institute of Biochemistry, Biocenter Goethe University Frankfurt Frankfurt 60438 Germany

**Keywords:** *Arabidopsis thaliana*, Cryo‐EM, manganese cluster, photosynthesis, Photosystem II structure, protein–water–cofactor interactions, water channels

## Abstract

- Photosystem II (PS II) is a large membrane‐bound protein complex that catalyses light‐driven water oxidation in plants and cyanobacteria. The structure of PS II is well studied in cyanobacteria; however, there are very few PS II structures from plants. The currently available plant PS II structures are comparatively low resolution and are frequently incomplete, that is, missing subunits or cofactors.
- We optimized the procedure for isolating PS II from *Arabidopsis thaliana* and employed cryo‐electron microscopy to generate a high‐resolution structure of an intact and oxygen‐evolving PS II from *Arabidopsis thaliana* at 2.44 Å resolution, which to date represents the highest resolution structure of PS II from higher plants.
- At this resolution, many water molecules within the PS II structure can be detected, including waters around the water‐splitting manganese cluster, the nonheme iron, and within the water/proton channels connecting these active sites to the protein exterior, allowing for the first detailed description of the water networks in *Arabidopsis thaliana* and comparison with the highly resolved cyanobacterial PS II.
- Our findings further the understanding of design principles of protein–water–cofactor interactions in photosynthetic water splitting, quinone reduction/exchange, and about the role of lipids at the interface between PS II and the light‐harvesting proteins.

## Introduction

Photosystem II (PS II) is a large protein complex located in the thylakoid membranes of cyanobacteria, algae and higher plants. PS II absorbs light energy and uses it to perform charge separation reactions that in turn drive the oxidation of water and the reduction of plastoquinone (see for instance reviews by Renger & Renger, 2008; Shevela *et al*., 2023). This reaction results in the production of molecular oxygen, as well as electrons and protons required for fixing CO_2_ into carbohydrates, making it indispensable for life on Earth.

**Fig. 1:**
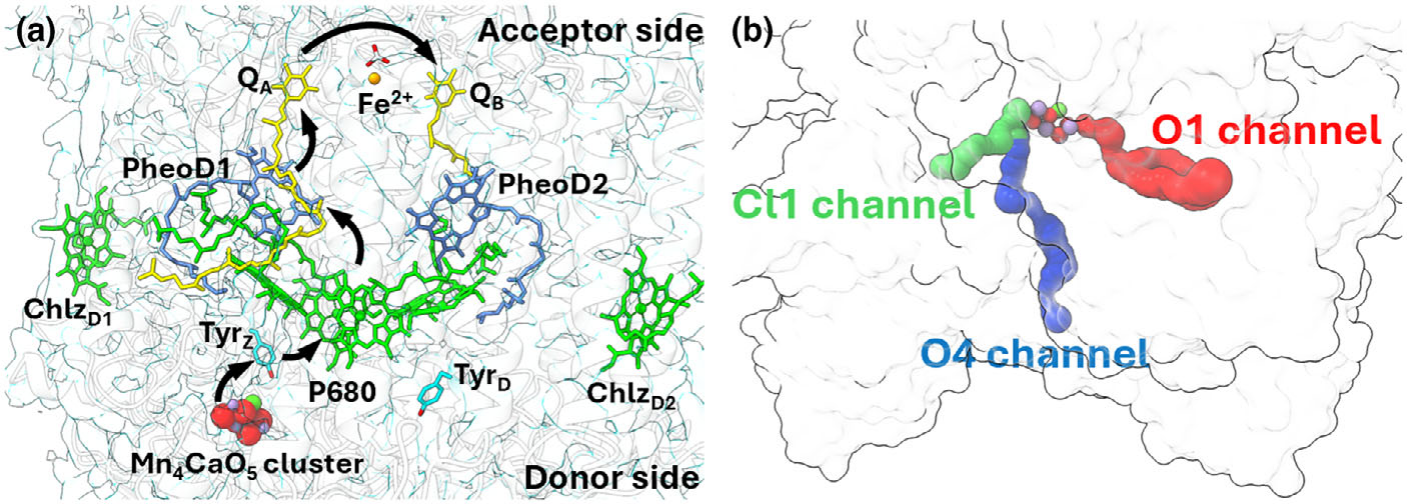
(a) Side view of Photosystem II (PS II) from *Arabidopsis thaliana* with essential cofactors. The Arabidopsis model is shown in white transparent cartoons with the Coulomb potential map in transparent blue. Important cofactors are highlighted in the structure: Mn‐cluster (red, purple and yellow spheres), Chl*a* (green), pheophytin molecules (blue), Q_A_ and Q_B_ molecules (yellow), nonheme iron (orange sphere), redox active tyrosines Tyr_Z_ and Tyr_D_ (cyan). Black arrows show the direction of electron transfer. The acceptor and donor side of PS II is labelled. (b) Channels which connect the Mn‐cluster to the protein exterior. The channels are shown in red (O1 channel), cyan (O4 channel) and green (Cl1 channel). The surface of PS II is shown faintly in white.

The water:plastoquinone redox process begins in the core of PS II with light induced charge separation between special chlorophyll (Chl) molecules, known as P680 and a pheophytin molecule (Pheo). This charge separation is stabilized by movement of the negative charge from Pheo^•−^ to the ‘acceptor side’ of PSII; initially to a nearby bound quinone (Q_A_) and then further on to a reversibly bound quinone (Q_B_). The positive charge on P680^•+^ is stabilized by electrons extracted from the manganese, calcium, oxygen cluster (Mn_4_CaO_5_ cluster; for short Mn‐cluster hereafter) via a nearby tyrosine (Y_Z_) on the ‘donor side’ of PS II (Fig. 1a). Four sequential oxidations of the Mn‐cluster build up its oxidizing potential, eventually leading to oxidation of water and the release of molecular oxygen and protons, while also restoring the electrons on the Mn‐cluster returning it to its ground state (Kok *et al*., 1970; Renger & Renger, 2008; Shevela *et al*., 2023). On the acceptor side, Q_B_ is reduced by two electrons and doubly protonated, before the reduced Q_B_ leaves PS II as plastoquinol (Q_B_H_2_) to be replaced by a new oxidized Q_B_ (Saito *et al*., 2013).

The ability of PS II from thermophilic cyanobacteria to readily form crystals has allowed cyanobacterial PS II structures to be obtained up to 1.89 Å resolution in *Thermosynechococcus vestitus* (previously known as *Thermosynechococcus elongatus*) or *Thermosynechococcus vulcanus* (Umena *et al*., 2011; Kern *et al*., 2014; Suga *et al*., 2015; Bhowmick *et al*., 2023). Recent improvements in single particle cryo‐electron microscopy (cryo‐EM) have also resulted in several near‐atomic resolution structures of PS II from cyanobacteria with resolutions up to 1.71 Å (Kato *et al*., 2021; Gisriel *et al*., 2022; Hussein *et al*., 2024). These high‐resolution structures have allowed comprehensive water modelling in cyanobacterial PS II structures, revealing water channels that connect the buried catalytic sites to the protein exterior (Sirohiwal & Pantazis, 2022). On the acceptors side, two permanent channels appear to connect Q_B_ to the exterior, namely Channels A and B, with a third channel, named Channel C, potentially opening upon the release of Q_B_ as plastoquinol (Umena *et al*., 2011; Sirohiwal & Pantazis, 2022; Sugo *et al*., 2022; Sugo & Ishikita, 2022; Hussein *et al*., 2024). On the donor side, three channels have been identified which provide the substrate waters and facilitate proton egress, namely the O1, O4 and the Cl1 channels (Fig. 1b). The O1 channel appears to be responsible for supplying substrate water to the Mn‐cluster, likely utilizing the recently discovered water wheel, a near planar pentagonal arrangement of five water molecules near the Mn‐cluster located within the O1 channel water network (Ibrahim *et al*., 2020; Bhowmick *et al*., 2023; Hussein *et al*., 2024; Li *et al*., 2024; Aydin *et* al., 2025). By contrast, the Cl1 channel has been shown to be crucial for proton release (Hussein *et al*., 2021; Allgöwer *et al*., 2022), while the O4 channel has been proposed as both a proton release and water delivery pathway (Kern *et al*., 2018; Suga *et al*., 2019; Kaur *et al*., 2024). However, to date the specific roles of the individual channels are still debated and the mechanisms on how they function are only beginning to emerge (Hussein *et al*., 2023).

Further insight into the function and design principles of the channels and water networks in PS II may be gained by inter‐species comparisons. Here, comparison between plant and cyanobacterial PSII is of special relevance, since evolutionary studies indicate that plants and cyanobacteria began to diverge from a common ancestor *c*. 1 billion years ago, following an endosymbiotic event (Cardona *et al*., 2019; De *et al*., 2024). The central subunits which perform charge separation and the electron transfer steps (mainly the D1 and D2 subunits) display a high conservation across all species; however, significant variability is found in the extrinsic proteins, which help to form the water channels, and the light‐harvesting antenna of higher plants and cyanobacteria.

One notable difference between plants and cyanobacteria is found in the functionally important and otherwise highly conserved region around the Mn‐cluster, where the D1:87 residue is an alanine in plants and an asparagine in cyanobacteria. Mutational studies have demonstrated that the identity of this residue can affect the binding of the chloride‐1 ion (Cl1; Banerjee *et al*., 2018), which is crucial for proton egress through the Cl1 channel (Boussac *et al*., 2012; Gates *et al*., 2023). Additionally, the identity of the residue at Position 87 affects the EPR signal from the Mn‐cluster in the presence of methanol (Retegan & Pantazis, 2017), which is regarded as a water analogue suitable for gaining information about water binding in PS II (Ho & Styring, 2008; Nöring *et al*., 2008).

The structures of plant PS II with an intact Mn‐cluster are available for Pea (*Pisum sativum*; 2.7 Å resolution) and Spinach (*Spinach oleracea*; 3.2 Å) and without the Mn‐cluster for Spruce (*Picea abies*; 2.8 Å) and *Arabidopsis thaliana* (2.7 Å, 3.0 Å; Caferri *et al*., 2025; Cao *et al*., 2018; Graça *et al*., 2021; Opatíková *et al*., 2023; Su *et al*., 2017; Van Bezouwen *et al*., 2017; Wei *et al*., 2016). However, even for relatively complete structures, such as the pea and spinach structures, the occupancy of some of the extrinsic subunits is extremely low (Supporting information Table S1). Thus, to date, there is very little information regarding the water/proton networks of plant PS II due to the lack of high‐resolution structures of intact, O_2_ evolving plant PSII, and therefore, only the shape of potential channels could be compared (Hussein *et al*., 2023).

Here, we present a fully intact, oxygen‐evolving C_2_S_2_‐PS II:LHC II supercomplex from *Arabidopsis thaliana* at a resolution of 2.44 Å. At this resolution, we were able to model waters within the water bonding networks of PS II. By comparing the water networks in our structure from *Arabidopsis thaliana* (hereafter Arabidopsis) to the recently published PS II structure from *Thermosynechococcus vestitus* (hereafter *T. vestitus*), we could investigate which waters within the water networks are conserved and what specific differences exist. We reveal the differences in the water network near the Mn‐cluster caused by the variation of the D1:87 residue between plants and cyanobacteria and show that otherwise the water networks are largely conserved between the species up to specific channel bottle necks, after which they may vary significantly. This unique analysis provides novel insight into the design principles and function of water channels in PSII.

## Materials and Methods

### Photosystem II core complex isolation and activity assay

*Arabidopsis thaliana* (L.) Heynh. (Columbia‐0) plants were grown on soil in a growth chamber for 8 wk at constant temperature (20°C) and humidity (70%), and day : night periods of 8 h : 16 h, light : dark at a light intensity of *c*. 125 μmol (photons) m^−2^ s^−1^ using full‐spectrum, white light LED lamps (Ra 95; LEDlife 50W Philipps, Amsterdam, the Netherlands). The growth of these plants complied with the local and national regulations (Dnr 4.6.20‐6365/15). The leaves of the plants were harvested after 16 h of dark, and BBY membranes (Berthold *et al*., 1981) were extracted as described previously (Chen *et al*., 2019).

PS II core complexes were prepared as follows: Berthold‐Babcock‐Yocum (BBY) membranes were washed and resuspended to a concentration of 1 mg ml^−1^ of Chl with wash buffer (25 mM MES pH 6.3, 10 mM NaCl, 5 mM CaCl_2_) and then diluted to 0.5 mg ml^−1^ with 0.6% (w/v) n‐dodecyl‐β‐maltoside (β‐DM) in wash buffer and left for 1 min, slowly stirring with a magnetic stirrer. Thereafter, the insoluble material was pelleted by first centrifuging at 16 000 ***g*** for 5 min, and then spinning the supernatant again at 20 000 ***g*** for 10 min. The supernatant of the second centrifugation was loaded onto the top of sucrose gradients which had been formed by the freeze‐thawing method (Luthe, 1983; 25 mM MES pH 6.3, 0.5 M sucrose, 1 M betaine, 10 mM NaCl, 5 mM CaCl_2_, 0.01% (w/v) β‐DM (Anatrace, Maumee, OH, USA)). Sucrose gradients were centrifuged at 141 000 ***g*** for 18 h, and the resulting bands were separated. Following washing and concentration of the individual bands, oxygen evolution measurements were made on the separated bands (Fig. S1) using a Clark‐type electrode (Hansatech, Pentney, UK). The bands were measured at a Chl concentration of 10 μg ml^−1^ using saturating light with 0.2 mM PPBQ (Phenyl‐p‐benzoquinone) and 1 mM K_3_FeCN_6_ added as electron acceptors (Fig. S1).

### Sample preparation and electron microscopy

C_2_S_2_ complexes were concentrated using 100 kDa molecular‐weight cut‐off spin filters (Merck, Darmstadt, Germany) and then washed several times with prefreezing buffer (50 mM MES pH 6.3, 20 mM CaCl_2_, 5 mM MgCl_2_, 0.6 M Betaine and 0.03% (w/v) β‐DM) before finally concentrating to a final concentration of 1.6 mg (Chl) ml^−1^. Four microlitres of sample was loaded onto glow‐discharged (30 s at 50 mA) Quantifoil grids R 1.2/1.3 Cu 300 (Quantifoil Micro Tools, Jena, Germany) in dim room light. The grids were then plunge frozen in liquid ethane using an FEI Vitrobot MkIV (Thermo Fisher Scientific) at 100% humidity, 4°C, and a blot force of −5, a wait time of 1 s and a blotting time of 5 s. Automated data collection was performed using the Epu software on a Titan Krios G2 transmission electron microscope operating at 300 keV (Thermo Fisher Scientific, Waltham, MA, USA) at the Umeå Core Facility for Electron Microscopy, a node of the Swedish National Cryo‐EM facility. The Titan Krios was equipped with Falcon 4i direct electron detector and a Selectris energy filter. In total, 16 450 movies were collected in EER format. The movies were collected at a pixel size of 0.7155 Å, a total dose of 40 electrons Å^−2^ and defocus values that ranged from −0.8 to −2.0. After data collection, the EER files were fractionated into 40 frames.

### Data processing

The dataset was processed using the CryoSPARC software (Punjani *et al*., 2017). Motion and contrast transfer function corrections were performed before template‐free picking was used to identify particles. 3054 062 particles were initially identified, before several rounds of 2D classification reduced the number of ‘good’ particles to 322 522. *Ab initio* 3D reconstruction was used to generate 6 different 3D volumes, which were then refined using heterogeneous refinement. The particles used to generate the best‐resolved class went through additional filtering using 2D classification and multi‐class *ab initio* 3D reconstruction until a final volume was generated using 72 301 particles and refined using homogeneous refinement and nonuniform refinement with imposed C2 symmetry. The final volume had an estimated global resolution of 2.44 Å, using the gold standard FSC (Fig. S2). Finally, the map was locally sharpened using the Phenix Autosharpen function (Liebschner *et al*., 2019).

### Model building

The model for spinach PS II (3JCU) was used as a starting point and manually fitted into the map using the UCSF ChimeraX software (v.1.7; Emsley *et al*., 2010). Coot (v.0.9.8.92; Meng *et al*., 2023) was used to perform the relevant mutations to change the *Spinacia oleracea* protein sequences to the corresponding *Arabidopsis thaliana* sequences. Coot was then used to refine the model to fit into the sharpened map. Several rounds of automatic fitting and manual fitting/checking were performed to optimize the fitting of the model into the map densities. Final refinement was performed using the real‐space refinement function in Phenix (Liebschner *et al*., 2019), with manual checking in Coot to confirm or correct the automatic refinement. All structures in the figures were prepared with ChimeraX or PyMOL (Schrodinger & Delano, 2020). Water molecules were modelled when there was a clear density at 1.29 Root‐mean‐squared‐deviation (RMSD) contour level within 3 Å of a potential hydrogen bonding group. Map and model statistics are provided in Tables S2–S5.

### Water channel calculations

The channels connecting the manganese cluster to the exterior of the protein were calculated using the Caver software (Chovancova *et al*., 2012; v.3.0.3), which is a plugin for PyMOL. For most of the channels, standard settings were used: probe radius 0.9, shell depth 4, shell radius 3 and clustering threshold 3.5. For the Cl1 channel, it was necessary to reduce the probe radius to 0.6.

### B‐factor analysis

The structure was refined using REFMAC5, implemented in Servalcat (Yamashita *et al*., 2021), with the two unsharpened half‐maps and the required mask file. Figures related to the analysis were prepared using PyMOL.

## Results

### C_2_S_2_ ‐type Photosystem II map and model

**Fig. 2:**
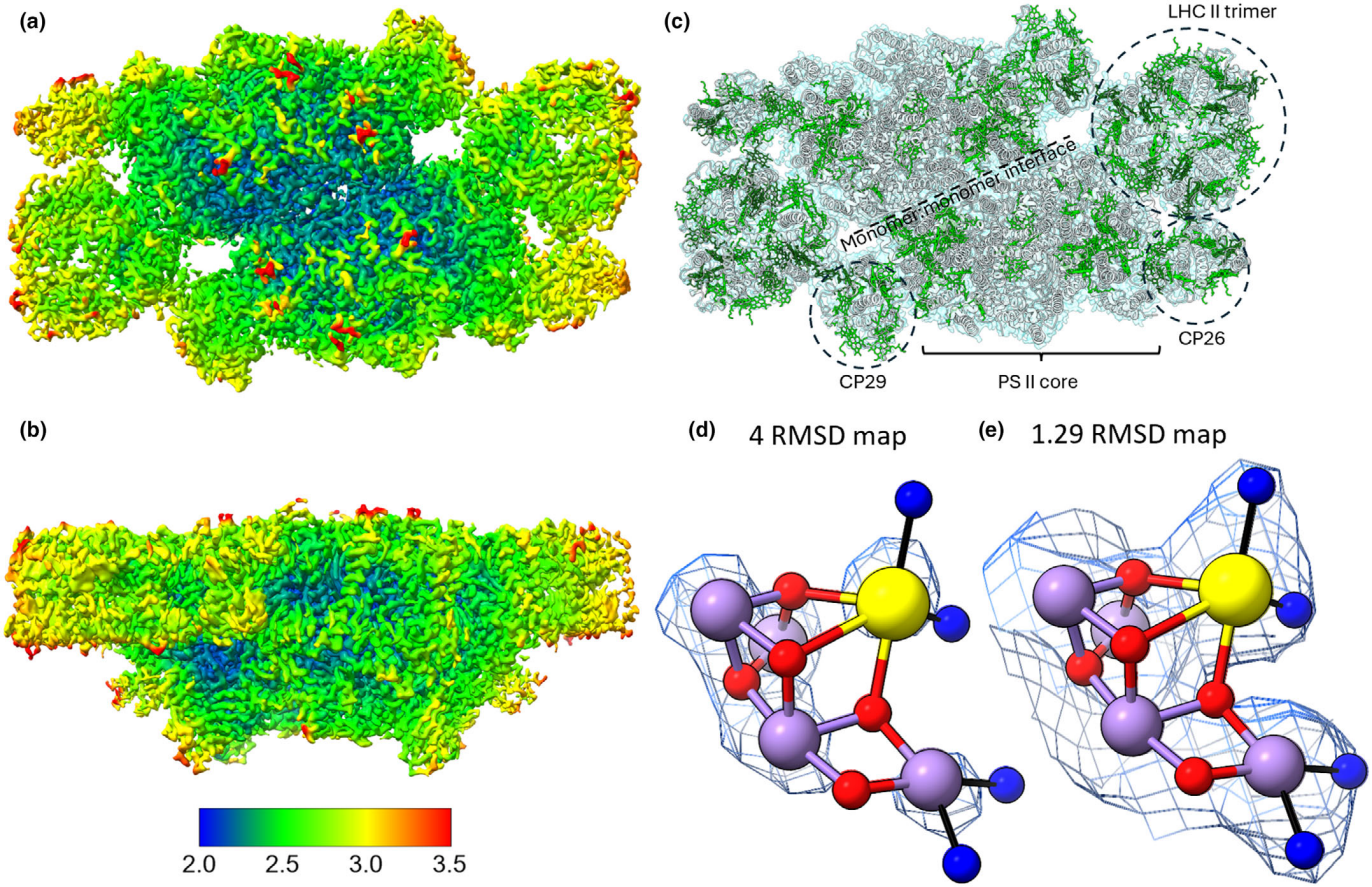
Side view (a) and top view (stromal side) (b) of the Coulomb potential map of Arabidopsis Photosystem II (PS II) coloured to show the local resolution. Bar indicating the colour key of the local resolution is shown below. (c) Top view (stromal side) of PS II from *Arabidopsis thaliana*. The Coulomb potential map is shown in transparent blue with the protein chains of the model shown in white. Chlorophyll molecules in the model are shown in light green (Chlorophyll *a*) or dark green (Chlorophyll *b*). The monomer:monomer interface is indicated with a black dashed line and labelled. For one of the monomers, the LHC II strongly bound trimer (s‐LHC II), CP26 and CP29 subunits are circled and labelled. (d) Fitting of the Mn‐cluster into the Coulomb potential map. The Mn‐cluster is shown as a collection of purple, red and yellow spheres to represent manganese, oxygen and calcium, respectively. The locally sharpened Coulomb potential map is shown as a mesh around the atoms at a contour level of 4.00 RMSD (d) or at a contour level of 1.29 RMSD (e).

The Coulomb potential map of the C_2_S_2_‐type PS II was generated from 72 301 particles with enforced C2 symmetry and reached a global resolution of 2.44 Å, ranging from 2.0 Å resolution in the core to 3.5 Å resolution in the flexible peripheral regions. This is the highest resolution reported so far for plant PS II. Additionally, this structure has high occupancy for the extrinsic subunits (Table S1); meanwhile, the map showed no density corresponding to the medium‐bound LHC II trimers in neither the 3D structure nor the 2D classes (Fig. 2a–c).

All PSII cofactors (Fig. 1a) are resolved, including the Mn and Ca ions of the Mn‐cluster (Fig. 2d) and its associated water ligands W1–W4 (Fig. 2e). The local resolution around the Mn‐cluster (2.15–2.22 Å) supports the reliable modelling of these atoms. The positions of the Mn and Ca ions were determined by centring them on the peak maxima of the Coulomb potential map and applying restraints to prevent movement during refinement. The resulting Mn–Mn and Mn–Ca distances are longer on average than comparable XFEL structures (Table S6; Kato *et al*., 2021; Kern *et al*., 2018), suggesting a reduction of the Mn ions during data collection (micrographs collected at 40 e^−^/Å^−2^). By contrast, the proteins appear to be mostly unaffected by the electron beam during data collection as the disulphide bond in PsbO protein, which is known to break at high electron doses (83 e^−^/Å^−2^) is intact in our structure (Kato *et al*., 2021; Fig. S3). Both the PsbO and PsbQ protein subunits may be present in two isoforms in Arabidopsis (Schubert *et al*., 2002). Fitting of both isoforms was performed (Fig. S4), and it was determined that PsbO1 and PsbQ1 showed the best agreement with the cryo‐EM potential map. No density for the PsbR, PsbS and PsbY subunits was observed (see the Discussion section).

Our structure includes 1206 modelled water molecules, compared with 1076 waters modelled in the pea structure (5XNL). This demonstrates the benefit of the increased resolution in expanding the water networks within PS II from land plants. In the current structure 1062 of the waters are associated with the PSII core complex, compared with 2405 in the PS II core complex from the cyanobacterium *T. vestitus* (Hussein *et al*., 2024) and 840 waters in the core complex from pea (5XNL).

### Water networks and structural differences at the PS II acceptor side

Waters modelled on the acceptor side of PS II (Fig. S5) form a network around the nonheme iron. This includes a water positioned between Q_B_, D1:Tyr246 and D1:His215 (water A:522, Fig. 3a). A water molecule in an equivalent position was also found in the PS II structure from *T. vestitus* (W63 in the 9EVX structure; Hussein *et al*., 2024). This finding suggests that this water may be part of a conserved re‐protonation pathway for the D1:His215 residue following donation of its proton to Q_B_, in line with previous theoretical predictions (Ishikita & Knapp, 2005; Saito *et al*., 2013; Sugo & Ishikita, 2022).

**Fig. 3:**
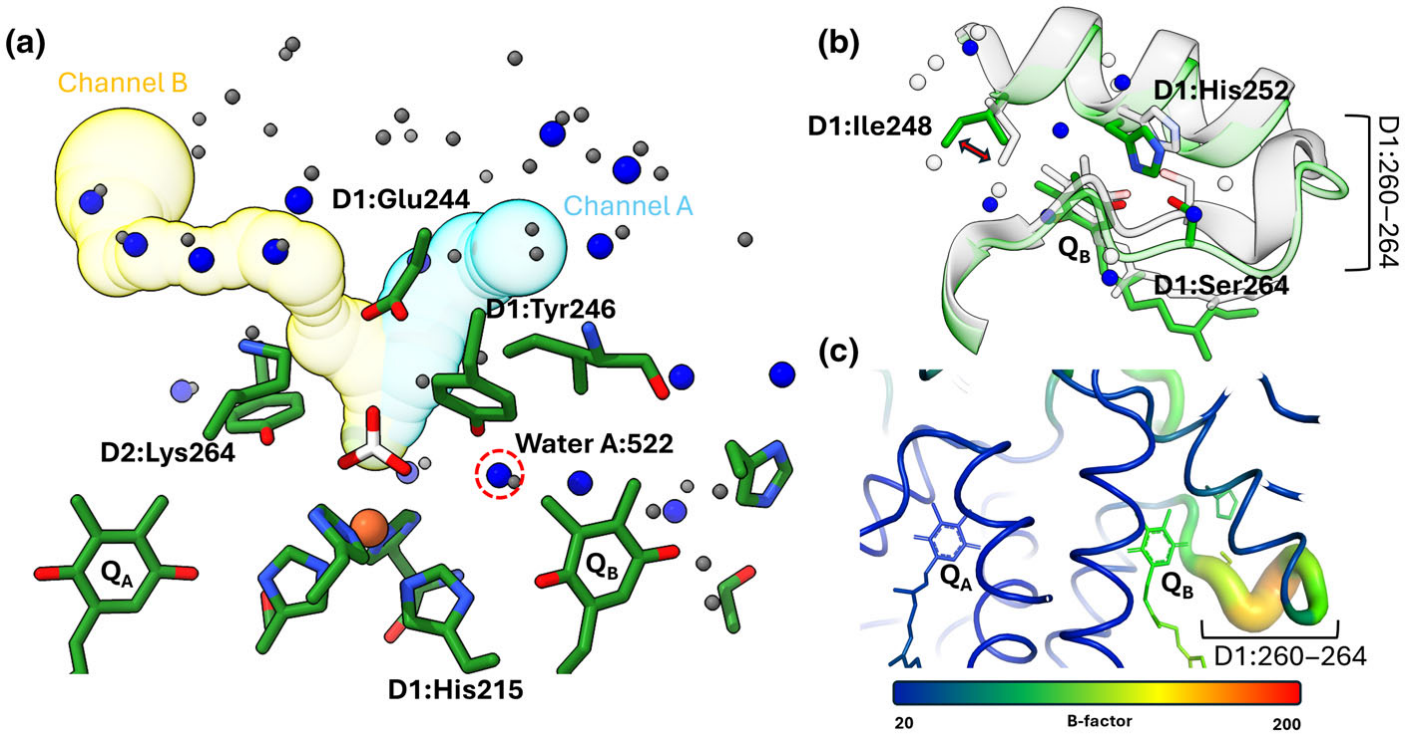
(a) Comparison of the water positions within 20 Å of the bicarbonate ligand to the nonheme iron in *Arabidopsis thaliana* (current structure) or *Thermosynechococcus vestitus* (9EVX). Waters from Arabidopsis are shown as blue spheres, and waters from *T*. *vestitus* are shown as grey spheres. For clarity, some water molecules and residues have been omitted. Selected residues are shown in green, with blue and red sections indicating nitrogen or oxygen atoms, respectively. Important residues are labelled. The bicarbonate ligand is shown in white, with red sections representing oxygen atoms. Water A:522 (corresponding to Water 63 in the 9EVX structure) plays a role in re‐protonation of D1:His215. The distances between water A:522 and D1:His215, D1:Tyr246 and the bicarbonate are as follows: 4.0, 2.3 and 3.4 Å, respectively. Channel A and Channel B were calculated in the Arabidopsis structure using the PyMOL plugin CAVER and are shown in yellow or cyan. (b) Comparison of the protein structure around the QB‐binding pocket in *Arabidopsis thaliana* (green, current structure) and *T. vestitus* (grey, 9EVX). The PsbA (D1) subunits have been aligned using the ChimeraX tool ‘Matchmaker’. The Q_B_, D1:His252, D1:Ser264 and D1:Ile.248 residues are shown as sticks. A double‐headed red arrow indicates the different side chain orientation of the D1:Ile248 residue in the two structures. (c) The acceptor side of PS II from the current structure. The structure is coloured to indicate the B‐factor. Light green to red colour gradient and a wider tube indicate regions with higher B‐factor. The bar is shown below the panel.

CAVER analysis of the acceptor side of the Arabidopsis model showed two channels connecting the bicarbonate ligand of the nonheme iron with the protein exterior (Fig. 3a). This is consistent with the acceptor side channels described previously in cyanobacteria, namely Channel A and Channel B (Sirohiwal & Pantazis, 2022; Hussein *et al*., 2024). Several of the acceptor side waters in the Arabidopsis structure were found within Channel B, indicating that Channel B holds a well‐regulated water network. All the waters modelled within Channel B had corresponding waters in the *T. vestitus* structure (Fig. 3a). This high level of conservation highlights the importance of this water network in both plants and cyanobacteria. By contrast, no waters were modelled within Channel A in the Arabidopsis structure. This suggests that waters in Channel A may have higher mobility and are therefore more difficult to detect.

Analysis of the acceptor side showed that the short D1:260‐264 region is not clearly resolved (Figs 3b, S6a) and features elevated *B‐*factors (Fig. 3c), indicating high flexibility/disorder in the Arabidopsis structure. Additionally, the D1:Ile248 side chain has a different conformation to the *T. vestitus* structure (9EVX; Figs 3b, S7). Analysis of the Q_B_‐binding pockets in other PS II structures also shows strong variability in the described region, despite identical sequences (Fig. S6b–e). We propose two reasons for this: (1) Difference map analysis shows a strong negative density at Q_B_ relative to Q_A_, confirming that a considerable fraction of the Arabidopsis particles lack Q_B_ (Fig. S8), while in the *T. vestitus* structure, this difference is much smaller (Hussein *et al*., 2024, supplemental). Occupancy refinement estimates the occupancy of Q_B_ to be *c*. 65%, relative to Q_A_. This lower Q_B_ occupancy in the Arabidopsis PSII may be expected, since the complexes were frozen in dim light, potentially leading to the reduction and release of Q_B_ in a fraction of the particles. Movement in the D1:260‐264 loop and a conformational change at D1:Ile248 have been calculated to occur in connection with the release/exchange of fully reduced Q_B_ (Sirohiwal & Pantazis, 2022; Sugo *et al*., 2022). It may thus be speculated that the structural variability in this region is caused by the averaging of PS II particles with either occupied or empty Q_B_ pockets, and therefore, different D1:260‐264 loop conformations; (2) The absence of the PsbR subunit may contribute to the reduced occupancy of Q_B_ and/or the structural disorder. This suggestion is supported by previous reports that PsbR plays a role in the stabilization of Q_B_ within the binding pocket (Shan *et al*., 2024) as well as the observation that in PsbR knockout mutants the electron transfer on the acceptor side is affected (Allahverdiyeva *et al*., 2007).

### Water networks around the Mn‐cluster

**Fig. 4:**
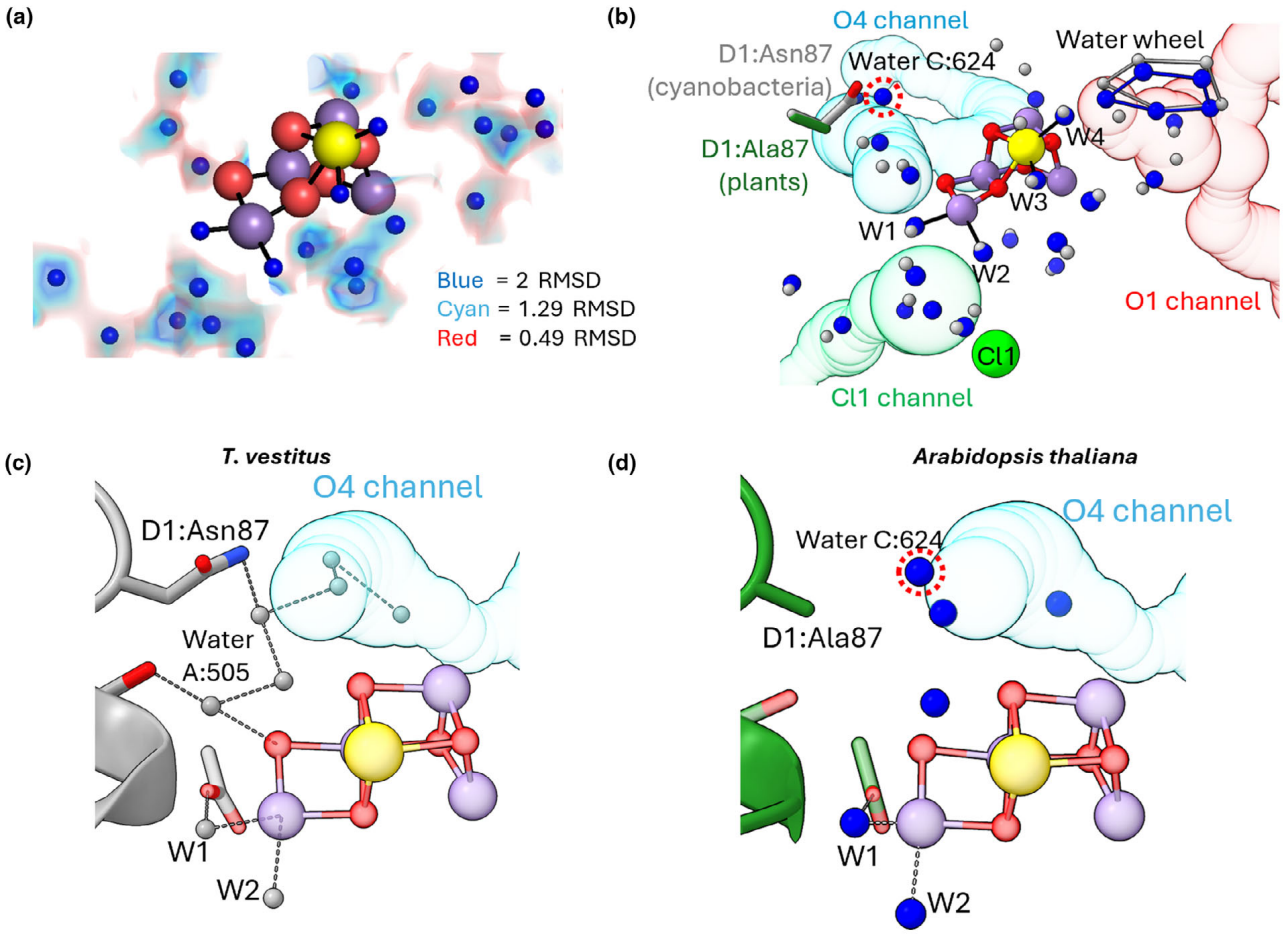
Comparison of the water networks around the Mn‐cluster. (a) Modelling of water molecules into electron density blobs around the Mn‐cluster in the Arabidopsis structure. The Coulomb potential densities from the locally sharpened map is shown for a radius of 1.5 Å around the modelled waters at 2 RMSD (blue), 1.29 RMSD (cyan) or 0.49 RMSD (red). Only waters within 9 Å of the Mn‐cluster are shown. (b) Comparison of the water network within 9 Å of the Mn‐cluster in *Arabidopsis thaliana* (current structure) or *Thermosynechococcus vestitus* (9EVX). The Mn‐cluster is shown as a cluster of red, purple or yellow spheres, representing O, Mn or Ca ions, respectively, and the Cl1 ion is shown as a lime‐green sphere. Black lines indicate bonds connecting specific water molecules to the Mn‐cluster. These bound waters are labelled as W1, W2, W3 and W4 (for Coulomb densities, see Fig. 2e). The C:624 water is also labelled and circled with a red dotted line. This water has no corresponding water in the *T. vestitus* structure (9EVX). The water wheel is indicated with lines connecting the constituent waters. The O1, O4 and Cl1 channels which lead to the exterior of the protein shown in red, blue and green, respectively (channels calculated using the CAVER plugin for PyMOL). The side chain of the alanine D1:87 residue in Arabidopsis is shown in green, while the asparagine D1:87 side chain from *T. vestitus* is shown in grey. (c, d) Comparison of the putative water network around the opening of the O4 channel in *T. vestitus* (c) and Arabidopsis (d). Waters are shown as grey spheres for *T. vestitus* and blue spheres for Arabidopsis. Water: A505 corresponds to water 19 in (Hussein *et al*., 2024) and is not shown in panel d due to very low charge density in the current Arabidopsis structure. The unique water found in Arabidopsis in place of the D1:Asn87 headgroup is circled with a red dotted line (Water C:624). This water is located 2.9 and 3.6 Å from the neighbouring waters A:644 and A:531, respectively. In both panels, the O4 channel is shown in light blue and the Mn‐cluster is shown as red, purple and yellow spheres.

In addition to the four water ligands of the Mn‐cluster (W1–W4), 23 water molecules were modelled in our structure within 9 Å of the catalytic site (Fig. 4a); for comparison, 19 waters were modelled in pea (5XNL), and 37 waters were modelled in *T. vestitus* (9EVX). For water modelling around the Mn‐cluster, we explored different contour levels. At first, 1.29 RMSD was used since below this level random noise begins to appear in locally sharpened maps outside the PSII complex. At this contour level, the map shows density for all the Mn‐cluster cavity water molecules, except for two of the five waters in the water wheel (Fig. 4a). Lowering the contour level to 0.49 RMSD displayed clear density also for the two remaining wheel water molecules, without the appearance of random noise in this region, which is due to higher local resolution in the core region (2.15–2.22 Å) compared with the periphery (Fig. 2a,b). This finding shows that the water wheel is conserved among species and supports the proposal that these flexible waters play a role in water oxidation, and that the water wheel might be involved in the insertion of a substrate water into the Mn‐cluster and/or the storage of a proton (Ibrahim *et al*., 2020; Bhowmick *et al*., 2023; Li *et al*., 2024; Aydin *et al*., 2025).

All the water molecules detected around the Mn‐cluster in Arabidopsis have an equivalent water in the *T. vestitus* structure, except for the water modelled near the D1:Ala87 residue (Chain C:624), which is in a similar position to the polar headgroup of the D1:Asn87 side chain of cyanobacteria (Fig. 4b). This change alters the water bonding network in the area near the O4 bridge of the Mn‐cluster (Fig. 4c,d); however, higher resolution and/or computational analysis is required to fully evaluate the details. We note that the Arabidopsis map displays a weak charge density at the same position as Water:A505 (also referred to as water 19 in Hussein *et al*., 2024; Fig. 4c). However, as the signal was very weak, no water was modelled in this position. This change in the water network around O4 is the only clear difference in the Mn‐cluster environments of plants and cyanobacteria; thus, it may be the cause of reported spectroscopic differences, such as variations in FTIR difference spectra and the presence/absence of the *g* = 4.1 S_2_ state EPR signal (Retegan & Pantazis, 2017; Corry & O'Malley, 2019).

Differences have also been observed in the Cl^−^ sensitivity in plants and cyanobacteria (Banerjee *et al*., 2018). Analysis of the Cl1 and Cl2 ions near the Mn‐cluster indicates that in these experimental conditions (i.e. Cl‐rich) the Cl1 and Cl2 ions both show high occupancy and are modelled in a similar position to other structures (Figs S9, S10).

### Water/proton channels to the Mn‐cluster

**Fig. 5:**
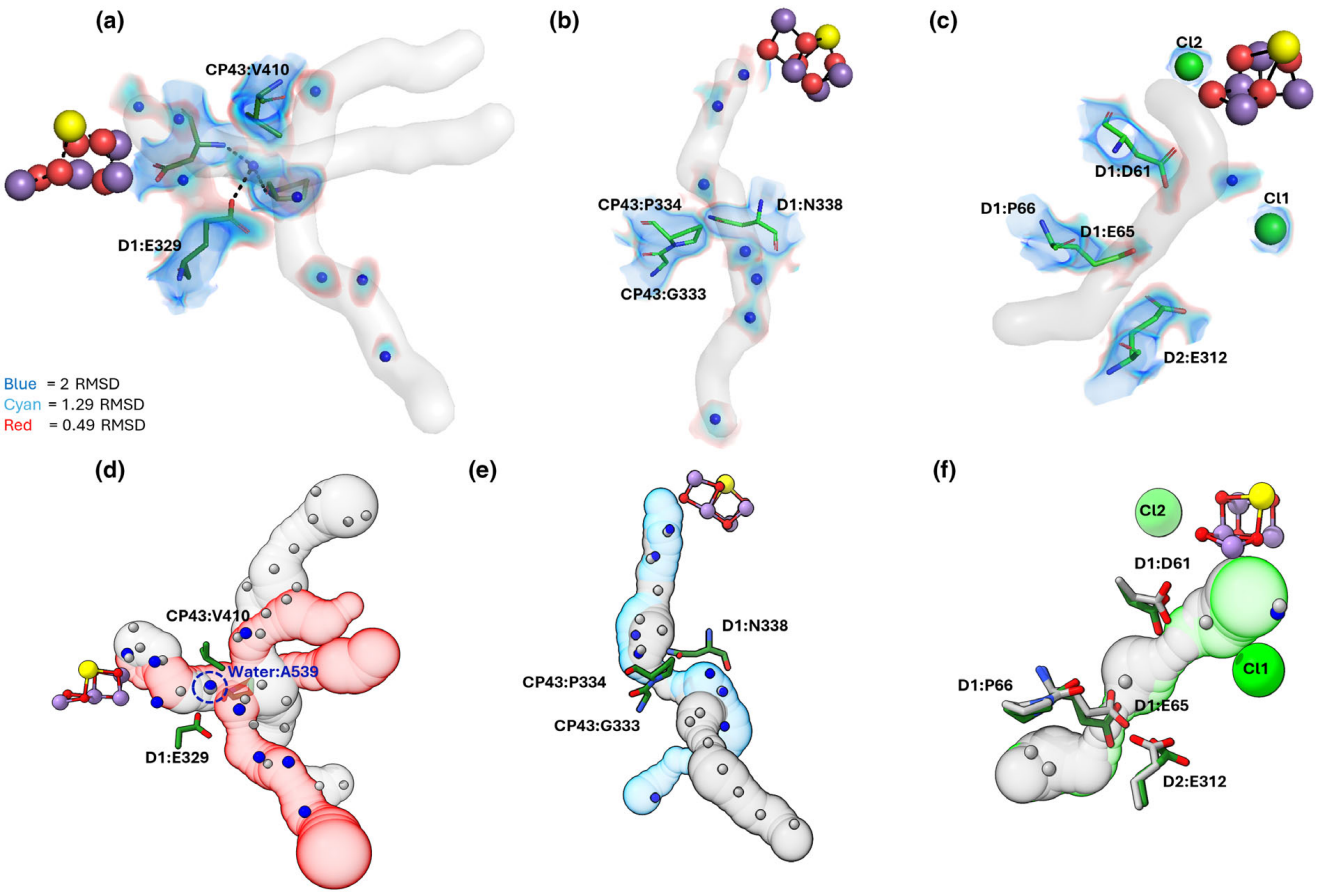
Water channels connecting the Mn‐cluster to the protein exterior. The modelling of waters within the O1 channel (a), the O4 channel (b) and the Cl1 channel (c). The Coulomb potential map is shown around the waters and bottleneck residues at contour levels of 2 RMSD (blue), at 1.29 RMSD (cyan) and at 0.49 RMSD (red). The channels are shown in faint grey. Bottleneck residues are shown for the O1 (a) and O4 (b) channels, and key proton‐transfer residues are shown in the Cl1 channel (c), as green sticks. The Cl1 and Cl2 atoms are shown as green spheres in (c). The Mn‐cluster is shown as a collection of red, purple and yellow spheres. Comparison of the channels in the Arabidopsis structure to the channels in *Thermosynechococcus vestitus* are shown in d–f. The O1 (d), O4 (e) and Cl1 (f) channels for the Arabidopsis structure are shown in red, blue and green, respectively, with the corresponding channels from *T. vestitus* (9EVX) shown in grey. (d) The residues which form the O1 channel bottleneck (D1:Glu329, D1:Pro340 and CP43:Val410) are shown in green, with blue and red sections indicating nitrogen or oxygen atoms, respectively. (e) The D1:Asn338 and CP43:Gly333; CP43:Pro334 residues which form the O4 channel bottleneck are shown in green. (f) Residues that are important for proton transfer in the Cl1 channel (D1:Asp61, D1:Glu65, D1:Pro66 and D2:Glu312) are shown as green sticks for Arabidopsis and grey sticks for *T. vestitus* (9EVX). The Cl1 atom is shown as a lime‐green sphere.

The O1 channel of Arabidopsis, for which we found nine well resolved waters (Fig. 5a), initially follows the same route as the O1 channel in *T. vestitus* until both pass a conserved bottleneck region that is occupied by one water molecule (Water A539; Fig. 5d; Hussein *et al*., 2021; Kern *et al*., 2018). However, after the bottleneck, the O1 channels of the two species diverge considerably (Fig. 5d). This divergence is in part due to the fact that Arabidopsis and *T. vestitus* have different extrinsic proteins; this is also seen in other species (Hussein *et al*., 2023). Importantly, the bottleneck water is also found in *T. vulcanus* (Li *et al*., 2024; 8IR5) and in *Pisum sativum* (5XNL; Su *et al*., 2017; Fig. S11). This suggests that this water is positionally restrained and functionally important, based on its consistent detection across diverse species.

The O4 channels in Arabidopsis and *T. vestitus* follow a similar path until they diverge when the O4 channel passes through the extrinsic proteins (Fig. 5b,e). Notably, the common, conserved part of the O4 channel is significantly longer and narrower than for the O1 channel. These highly conserved characteristics of the O4 channel, particularly the extended region with a precisely tuned water chain, are consistent with its proposed role as a proton egress channel in both cyanobacteria and higher plants, which may support H^+^ release during the S_0_‐S_1_ transition of the Mn‐cluster (Takaoka *et al*., 2016; Shimizu *et al*., 2018; Shimada *et al*., 2020; Hussein *et al*., 2021).

The shape of the Cl1 channel is highly similar between Arabidopsis and *T. vestitus* (Fig. 5c,f), including conserved positions for the critical proton‐transfer residues and water molecules (Hussein *et al*., 2021; Allgöwer *et al*., 2022; Mäusle *et al*., 2024). This suggests that the function of the Cl1 channel for gated proton release, which has been described for cyanobacteria and, based on the structural similarity, appears to be conserved in higher plants. Only one water molecule could be modelled in the Cl1 channel of Arabidopsis (Fig. 5c).

### Lipid cofactors

In addition to water networks, the enhanced resolution of this PS II structure from higher plants allows us to examine features unique to higher plants, such as the interface between the PSII core and the antenna complexes. In higher plants, the antenna complex is made up of the membrane‐bound light‐harvesting complex II (LHC II) trimers, along with linker proteins (Arshad *et al*., 2022) instead of the soluble phycobilisomes found in cyanobacteria (Zheng *et al*., 2021). At the PS II‐LHC II trimer interface, the lipids digalactosyl diacylglycerol (DGD; LHCB2.2 [ChainY,y]:203) and sulfoquinovosyl diacylglycerol (SQD; PsbW:202) were modelled (Fig. S12). These lipids presumably help to anchor the strongly bound LHC II trimers to the PSII complex in higher plants. Previous Arabidopsis structures had modelled these lipids as dipalmitoyl‐phosphatidyl‐glycerole molecules (LHG; Graça *et al*., 2021; Caferri *et al*., 2025), however, with the improved resolution of this structure we were able to confidently identify these lipids as SQD and DGD. Interestingly, structures of PS II from other higher plant species either have different lipids modelled in this position (pea; 5XNL, spruce; 8C29) or no lipids modelled (spinach; 3JCU). The differences in the cryo‐EM maps from Arabidopsis, Pea and Spruce at this position are significant enough to suggest that the identity of these lipids may vary between species or with growth/light conditions (Figs S13, S14). We speculate that the identity of these lipids may fine‐tune the light adaptation mechanisms in plants by modulating the threshold at which these complexes dissociate due to high light conditions (Kouřil *et al*., 2013; Grinzato *et al*., 2020). We note that further experiments are required to test this hypothesis.

In the structure of PS II from spruce (8C29), additionally an α‐tocopherol was modelled in this interface. We found an unmodelled density at a similar position (Fig. S15); however, the fit was not perfect, and the filling of this density by other molecules such as detergents (dodecyl‐β‐maltoside) could not be excluded. Two additional sites of α‐tocopherol binding have been predicted in PS II from Arabidopsis (Kumar *et al*., 2021), one in the vicinity of Pheo_D1_, near D1:Glu130, and the other near the nonheme iron. We found no unmodelled densities that could fit an α‐tocopherol in either of these locations.

Finally, we also observed a large unmodelled density at the monomer:monomer interface which provided a good yet not unique fit for a β‐carotene molecule (Fig. S16); thus, this potential β‐carotene was not included in the model.

## Discussion

The structure presented here is the highest resolution structure of PS II from plants and improves on the previous Arabidopsis PS II structures (7OUI, RLK4, RLK5; Caferri *et al*., 2025; Graça *et al*., 2021) as it has a higher resolution and includes the O_2_‐evolving Mn‐complex as well as all the extrinsic and nearly all low molecular weight.

No density was observed for the PsbR subunit. PsbR has recently been observed for the first time in a C_4_S_4_M_2_ PS II megacomplex from *Arabidopsis thaliana* and its function was assigned to supporting the formation of PSII megacomplexes and contributing to connections between opposite thylakoid membranes (Shan *et al*., 2024). In line with this, PsbR was located in the enclosed area found between the pair of PS II dimers, but not at the equivalent positions at the periphery of the respective PS II dimers, suggesting that PsbR could easily be removed when exposed to detergent during isolation of the PS II‐LHC II C_2_S_2_ supercomplexes.

The PsbS and PsbY subunits were also not identified in our structure. PsbS is specific to plants, while in cyanobacteria PsbY is located on the periphery of PS II and appears to interact with cytochrome b559 (Hussein *et al*., 2024). Both PsbS and PsbY have been detected with Western blots in plant PS II preparations (Funk *et al*., 1995; Nield *et al*., 2000; Von Sydow *et al*., 2016); however, neither subunit has yet been observed in plant PS II structures.

The improvement from our previous Arabidopsis structure (7OUI) was achieved by optimizing the isolation procedure, which includes omitting digitonin and lowering the pH during PS II extraction. The functionality of the complex was established by oxygen evolution measurements (Fig. S1). It is interesting to note that the PS II particles recently used for the spruce and Arabidopsis structures (Caferri *et al*., 2025; Opatíková *et al*., 2023; 8C29, RLK4 and RLK5) were isolated at pH 7.5 or higher and lacked PsbJ, PsbP, PsbQ and the Mn‐cluster, despite the absence of digitonin, suggesting that pH may be more important than the choice of detergent for retaining the extrinsic subunits and the Mn‐cluster (Beauregard *et al*., 1988). The PsbP and PsbQ subunits have been previously modelled in the Spinach structure (3JCU), where the PS II particles were isolated at pH 7.5 and the Pea structure (5XNL), where the particles were initially solubilized at pH 7.5 before being purified at pH 5.7. Further analysis of these structures and their accompanying maps (emd‐6617, emd‐6741), showed that the spinach structure lacked electron density for the PsbP and PsbQ subunits and the Pea structure lacked electron density for the PsbQ subunit, indicating very low occupancy. This was demonstrated with the low atom inclusion and Q‐scores for these subunits (Table S1). This is presumably due to the loss of a large fraction of these extrinsic proteins due to high pH during isolation. Comparison of the current structure to the previous Arabidopsis structure which lacks PsbP and PsbQ (7OUI) shows changes in loop regions of CP43 and PsbO (Fig. S17), suggesting that the PsbP and PsbQ subunits play a role in stabilizing these specific loops.

Around the Q_B_‐binding site (D1:247‐267) differences were detected between this Arabidopsis structure and the *T. vestitus* structure, despite the sequence being identical in these organisms. We hypothesized that this difference is due to different Q_B_ occupancy in the two structures due to different light conditions during freezing. This supports previous theoretical calculations which propose that loop movements occur when the Q_B_ site is vacated (Sirohiwal & Pantazis, 2022; Sugo *et al*., 2022). However, it may also be caused by the absence of PsbR (Shan *et al*., 2024).

The resolution reached in this structure enables the first detailed comparison of the water networks around the Mn‐cluster in plant and cyanobacterial PSII. The water molecules in the inner cavity around the Mn‐cluster are demonstrated here to have overall highly conserved positions, despite a local deviation near Asp61 and the O4 bridge of the Mn‐cluster due to the D1:Asn/Ala87 sequence difference. This reinforces the emerging picture of the important role of these waters in the water oxidation reaction (Suga *et al*., 2019; Ibrahim *et al*., 2020; Hussein *et al*., 2021; Allgöwer *et al*., 2022; Bhowmick *et al*., 2023; Greife *et al*., 2023; Li *et al*., 2024; Mäusle *et al*., 2024; Aydin *et al*., 2025; Flesher *et al*., 2025), and the local deviation may explain known differences between plants and cyanobacteria regarding, for example, S2 state EPR signals (Banerjee *et al*., 2018; Corry & O'Malley, 2019).

The conservation of the donor side water/proton channels differs depending on the channel. The overall shape of the Cl1 channel is fully conserved between plants and cyanobacteria, including the positions of the amino acids identified to be involved in proton gating (Hussein *et al*., 2021; Bhowmick *et al*., 2023). However, the current structure resolves only one water molecule so that a more detailed comparison of the Cl1 channel must await higher resolution structures of Arabidopsis. While the entry into the O4 channel varies due to the D1:Asn/Ala87 sequence difference, the homology of this narrow channel is nearly perfect in both shape and water positions up to a branching point close to the protein surface. This well‐ordered single file of water molecules has previously been proposed to be a proton egress pathway, complementary to the Cl1 channel (Saito *et al*., 2013; Takaoka *et al*., 2016; Shimizu *et al*., 2018; Shimada *et al*., 2020). We propose that the high degree of conservation suggests a compulsory function, most likely to facilitate proton release during the S_0_‐S_1_ transition where the Cl1 channel may not operate. This proposal is in line with computation evidence showing that access to the Cl1 channel may open and close due to electrostatic interactions (Allgöwer *et al*., 2022). The Mn‐cluster is uncharged in its S_0_ oxidation state, as compared to having a positive charge in S_2_ and S_3_ (Klauss *et al*., 2012). Thus, in the S_0_ state, the absence of the positive charge may prevent the opening of the Cl1 channel gate, resulting in proton export through the O4 channel instead.

The O1 channel was previously proposed to be responsible for water delivery to the Mn‐cluster (Suga *et al*., 2019; Ibrahim *et al*., 2020; Hussein *et al*., 2023; Aydin *et* al., 2025). Our analysis shows that the O1 channel is conserved between the Mn‐cluster and the bottleneck region, including the water wheel region, but diverges considerably from the *T. vestitus* O1 channel from this bottleneck to the protein exterior. This is consistent with the O1 channel being a water delivery channel as from the outside up to the bottleneck the only requirement would be that water can freely reach the bottleneck, and thus, the channels can vary dramatically between species without functional consequences. It has been proposed that this bottleneck region can act as a ‘water valve’ that can ‘open’ and ‘close’ (increase/decrease water flux) at desired times during water oxidation, regulating water access to the Mn‐cluster (Sakashita *et al*., 2017; Suga *et al*., 2019). The mechanism for this has been hypothesized to involve the Y_Z_, D1:H190, D1:Q165 triad (Li *et al*., 2024), allowing the water valve to open for rapid water entry during the water insertion reactions in the S_2_‐S_3_ and S_3_‐S_0_ transitions (Fig. S18; Bhowmick *et al*., 2023; Hussein *et al*., 2021; Li *et al*., 2024), while being otherwise closed to restrict water/proton exchange and thereby stabilizing the critical protein–water–cofactor network and the water‐oxidizing complex in PS II.

### Conclusion

The improved resolution and intactness of our PS II structure from Arabidopsis allowed us to map large parts of its water networks and, thereby, to compare for the first time in detail the water networks of higher plants and cyanobacteria. This analysis furthers our understanding of PS II function, as conserved features of the water networks are likely to be functionally important, given that they have been retained for *c*. 1 billion years, since plants and cyanobacteria diverged during evolution.

## Competing interests

None declared.

## Author contributions

WPS and JM designed the study. JF and ATG isolated the PSII complexes. JF and ATG performed the SPA cryo‐EM data collection with support of MH. JF and ATG performed the SPA cryo‐EM data processing. JF and ATG performed the model building and refinement with contributions from RH. JF performed the CAVER analysis with contributions from AOA. JF and RH made the figures. JF and JM wrote the manuscript with input from all authors. JF and ATG contributed equally to this work.

## Disclaimer

The New Phytologist Foundation remains neutral with regard to jurisdictional claims in maps and in any institutional affiliations.

## Supporting information


**Fig. S1** Sucrose gradient separation and O_2_‐evolution activity of solubilized PS II particles.
**Fig. S2** Overview of CryoSPARC data processing.
**Fig. S3** A frequently damaged disulphide bond in the PsbO protein.
**Fig. S4** Comparison of the different isoforms of PsbO and PsbQ.
**Fig. S5** Modelling of water molecules on the acceptor side of PS II.
**Fig. S6** Comparison of the Q_B_‐binding pocket in various species.
**Fig. S7** Modelling of the D1:Ile248 residue.
**Fig. S8** Difference map analysis at the Q_B_ site.
**Fig. S9** Coulomb potential density around chloride ions.
**Fig. S10** Chloride ion locations in the Arabidopsis, *Thermosynechococcus vestitus*, pea and spinach structures.
**Fig. S11** Hydrogen bonding interactions at the O1 channel bottleneck.
**Fig. S12** Lipids found at the PS II core:LHC II interface.
**Fig. S13** Modelling of lipids at the PS II core:LHC II interface in various species (1/2).
**Fig. S14** Modelling of lipids at the PS II core:LHC II interface in various species (2/2).
**Fig. S15** Possible fitting of an α‐tocopherol molecule.
**Fig. S16** Possible fitting of a β‐carotene molecule.
**Fig. S17** Donor side differences in Arabidopsis model with and without PsbP and PsbQ subunits.
**Fig. S18** Connections between the Y_z_, D1:165, D1:190 triad and the putative water valve.
**Table S1** Atom inclusion and Q‐scores for the Arabidopsis, spinach and pea structures.
**Table S2** Summary of cryo‐electron microscopy data collection parameters.
**Table S3** Extent of modelling for each individual subunit.
**Table S4** Number and abbreviations of PS II cofactors.
**Table S5** Ligands within individual subunits.
**Table S6** Mn‐cluster distances in this structure and other PS II structures.
Please note: Wiley is not responsible for the content or functionality of any Supporting Information supplied by the authors. Any queries (other than missing material) should be directed to the *New Phytologist* Central Office.

## Data Availability

Model and map data are deposited on pbd (https://www.rcsb.org/) under the accession code 9I4T.
