# Supplementary material for: The structure of intact and active Photosystem II from Arabidopsis thaliana at 2.44 Å resolution
Source: New Phytol. 2026 Apr 10;250(5):3014–25. doi: 10.1111/nph.71085 (PMC13150307; doi:10.1111/nph.71085)
Supplement: Supplementary file 1 — Fig. S1 Sucrose gradient separation and O2‐evolution activity of solubilized PS II particles. Fig. S2 Overview of CryoSPARC data processing. Fig. S3 A frequently damaged disulphide bond in the PsbO protein. Fig. S4 Comparison of the different isoforms of PsbO and PsbQ. Fig. S5 Modelling of water molecules on the acceptor side of PS II. Fig. S6 Comparison of the QB‐binding pocket in various species. Fig. S7 Modelling of the D1:Ile248 residue. Fig. S8 Difference map analysis at the QB site. Fig. S9 Coulomb potential density around chloride ions. Fig. S10 Chloride ion locations in the Arabidopsis, Thermosynechococcus vestitus, pea and spinach structures. Fig. S11 Hydrogen bonding interactions at the O1 channel bottleneck. Fig. S12 Lipids found at the PS II core:LHC II interface. Fig. S13 Modelling of lipids at the PS II core:LHC II interface in various species (1/2). Fig. S14 Modelling of lipids at the PS II core:LHC II interface in various species (2/2). Fig. S15 Possible fitting of an α‐tocopherol molecule. Fig. S16 Possible fitting of a β‐carotene molecule. Fig. S17 Donor side differences in Arabidopsis model with and without PsbP and PsbQ subunits. Fig. S18 Connections between the Yz, D1:165, D1:190 triad and the putative water valve. Table S1 Atom inclusion and Q‐scores for the Arabidopsis, spinach and pea structures. Table S2 Summary of cryo‐electron microscopy data collection parameters. Table S3 Extent of modelling for each individual subunit. Table S4 Number and abbreviations of PS II cofactors. Table S5 Ligands within individual subunits. Table S6 Mn‐cluster distances in this structure and other PS II structures. Please note: Wiley is not responsible for the content or functionality of any Supporting Information supplied by the authors. Any queries (other than missing material) should be directed to the New Phytologist Central Office. [file NPH-250-3014-s001.pdf]

New Phytologist **Supporting Information**

Article title: **The structure of intact and active Photosystem II from *Arabidopsis thaliana* at 2.44 Å resolution**

Authors: Jack Forsman, André Graça, Abuzer Orkun Aydin, Michael Hall, Rana Hussein, Wolfgang P. Schröder, Johannes Messinger

Article acceptance date: 15 February 2026

The following Supporting Information is available for this article:

Fig. S1 Sucrose gradient separation and O_2-evolution activity of solubilised PS II particles.

Fig. S2 Overview of CryoSPARC data processing.

Fig. S3 A frequently damaged disulfide bond in the PsbO protein.

Fig. S4 Comparison of the different isoforms of PsbO and PsbQ.

Fig. S5 Modelling of water molecules on the acceptor side of PS II.

Fig. S6 Comparison of the Q_B binding pocket in various species.

Fig. S7 Modelling of the D1:Ile248 residue.

Fig. S8 Difference map analysis at the Q_B site.

Fig. S9 Coulomb potential density around chloride ions.

Fig. S10 Chloride ion locations in the Arabidopsis, *T. vestitus*, pea and spinach structures.

Fig. S11 Hydrogen bonding interactions at the O1 channel bottleneck.

Fig. S12 Lipids found at the PS II core:LHC II interface.

Fig. S13 Modelling of lipids at the PS II core:LHC II interface in various species (1/2).

Fig. S14 Modelling of lipids at the PS II core:LHC II interface in various species (2/2).

Fig. S15 Possible fitting of an α-tocopherol molecule.

Fig. S16 Possible fitting of a β-carotene molecule.

Fig. S17 Donor side differences in Arabidopsis model with and without PsbP and PsbQ subunits.

Fig. S18 Connections between the Y_z, D1:165, D1:190 triad and the putative water valve.

Supplementary table 1 Atom inclusion and Q-scores for the Arabidopsis, spinach and pea structures.

Supplementary table 2 Summary of cryo-electron microscopy data collection parameters.

Supplementary table 3 Extent of modelling for each individual subunit.

Supplementary table 4 Number and abbreviations of PS II cofactors.

Supplementary table 5 Ligands within individual subunits.

Supplementary table 6 Mn-cluster distances in this structure and other PS II structures**.**

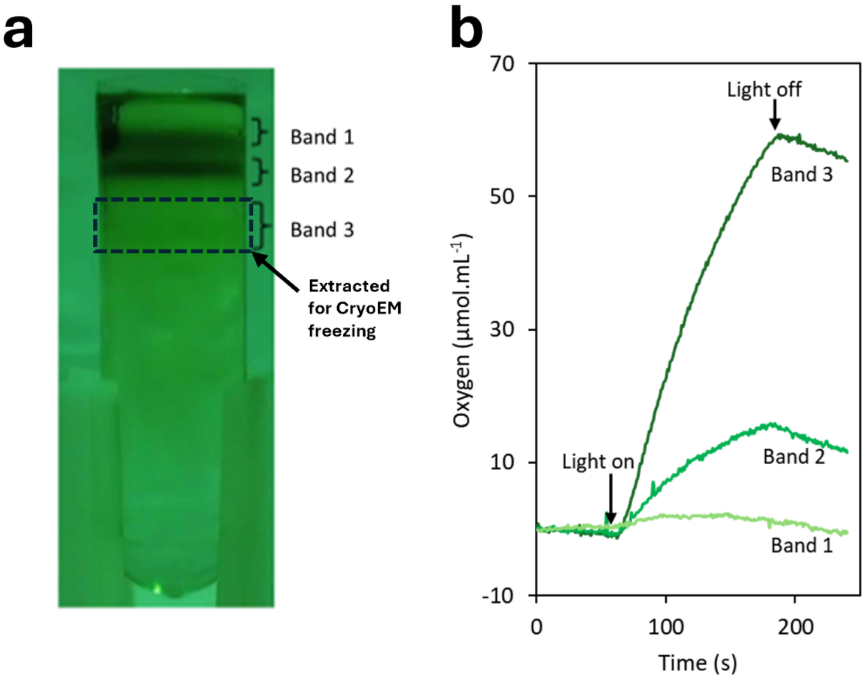

Fig S1: a. 0.5 M sucrose gradient used to separate the n-dodecyl-β-maltoside (β-DM) solubilised protein complexes from *Arabidopsis thaliana* BBY membranes. The band which was extracted for cryo-EM freezing and analysis is indicated with a dashed black box **b.** Oxygen evolving activity of the different bands collected from the sucrose gradient. Oxygen evolution was measured at 10 µg Chl mL^{-1} and supported using PPBQ and $FeCN_6$ as electron acceptors. The initial rates of oxygen evolution for Band 1, Band 2 and Band 3 are 20, 75 and 240 µmol O_2 (mg Chl)$^{-1}$ h^{-1}, respectively. Band 3 was frozen onto cryo-EM grids for cryo-EM analysis.

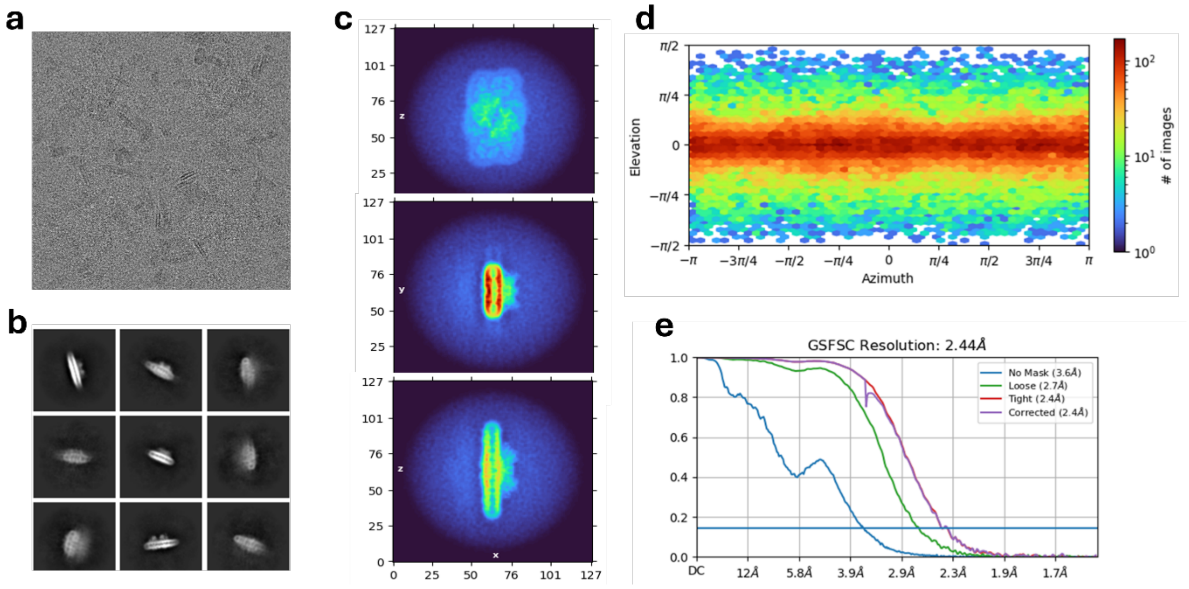

Fig S2: Cryo-electron microscopy workflow. **a.** Example of one of the 16,450 micrographs collected. Micrographs were collected at 215,000 X magnification. **b.** Example of some of the 2D classes used to separate the 3,054,062 particles extracted from the micrographs into 322,522 “good particles”. **c.** Example of the best class from *Ab initio* reconstruction. This class was formed from 72,301 particles. **d.** Distribution of the orientation of the particles used to generate the best class from *ab initio* reconstruction. **e.** Gold standard FSC curve for the refined maps from the best class of *ab initio* reconstruction (**c.**).

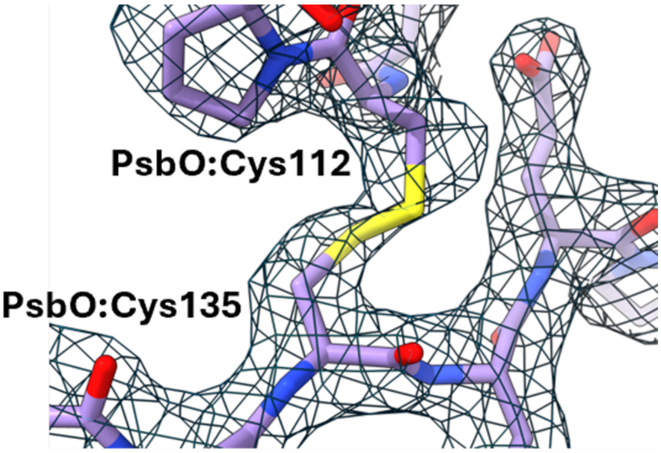

Fig S3: A frequently damaged disulfide bond in the PsbO protein with the associated cryo-EM map density. This disulfide bond was largely intact in this structure. PsbO residues are shown as purple sticks with oxygen atoms shown in red, nitrogen atoms shown in blue, sulfur atoms are shown in yellow. The Coulomb potential map of the locally sharpened cryo-EM map at a contour level of 1.5 RMSD is shown as a black mesh.

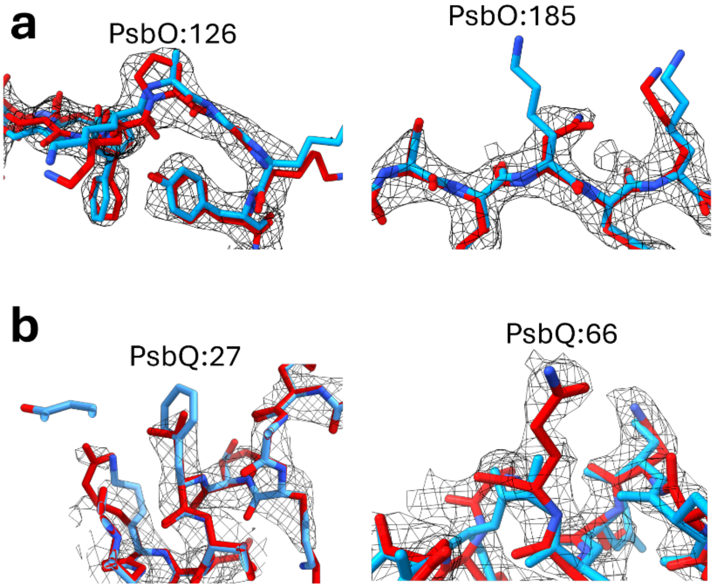

Fig. S4: Comparison of the different isoforms of PsbO and PsbQ. Atomic models of PsbO1 and PsbQ1 (red) were aligned with atomic models of PsbO2 and PsbQ2 (blue) and fit into the locally sharpened Coulomb potential map at a contour level of 1.29 RMSD. **(a)** Comparison of the two PsbO isoforms at position 126 and 185 shows that the residues of PsbO1 fit the Coulomb potential map better than PsbO2. **(b)** Comparison of the PsbQ isoforms at position 27 and 66 shows that the residues of PsbQ1 fit the Coulomb potential map better than PsbQ2.

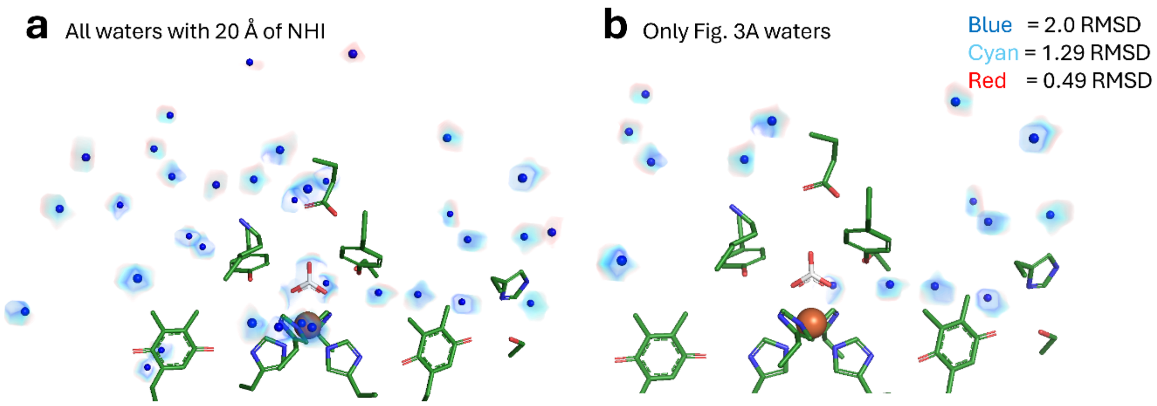

Fig. S5: Modelling of water molecules into electron density blobs on the acceptor side of the Arabidopsis structure. **(a)** All of the waters within 20 Å of the non-heme iron are shown blue spheres. The coloumb potential density from the locally sharpened map is shown for a radius of 1.3 Å around the modelled waters at 2 RMSD (blue), 1.29 RMSD (cyan) or 0.49 RMSD (red). Q_A, Q_B and the important residues D1:H215, E244,Y246, H252, S264, S268, H272, D2: H214, Y244, K268, H268 are shown as green sticks, with red and blue sections indicating, oxygen and nitrogen, respectively. **(b)** The same as panel **(a)** but only showing the waters which were visible in Fig 3a. Several waters were omitted in Fig. 3a for clarity.

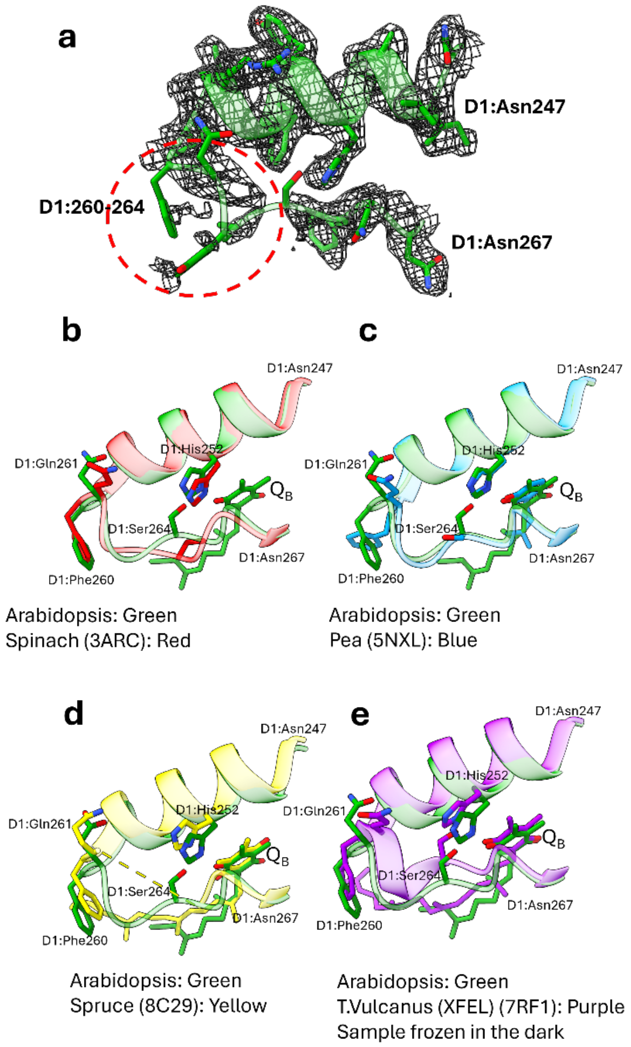

Fig. S6: Comparison of the protein structure around the Q_B binding pocket in various species. **(a)** The fitting of the D1:247-267 region in the current Arabidopsis model into the Coulomb potential map. The model is shown in green with red and blue sections indicating oxygen and nitrogen atoms, respectively. The Locally sharpened map at a contour level of 1.29 RMSD is shown as a black mesh. The D1:260-264 region which lacks density is circled. The PsbA (D1) subunit was aligned to the D1 subunit in other species using the ChimeraX tool “Matchmaker”. The Q_B, D1:His252, D1:Phe260, D1:Gln261 and D1:Ser264 residues are shown as sticks. Arabidopsis thaliana (green, current structure) is compared to **b.** Spinacia oleracia (red, 3JCU), **c.** Pisum sativum (blue, 5XNL), **d.** Pisia abies (yellow, 8C29), **e.** Thermosynechococcus vulcanus (XFEL) (purple, 7RF1). In panel **d**, the dashed yellow line indicates where residues were not modelled due to low map resolution.

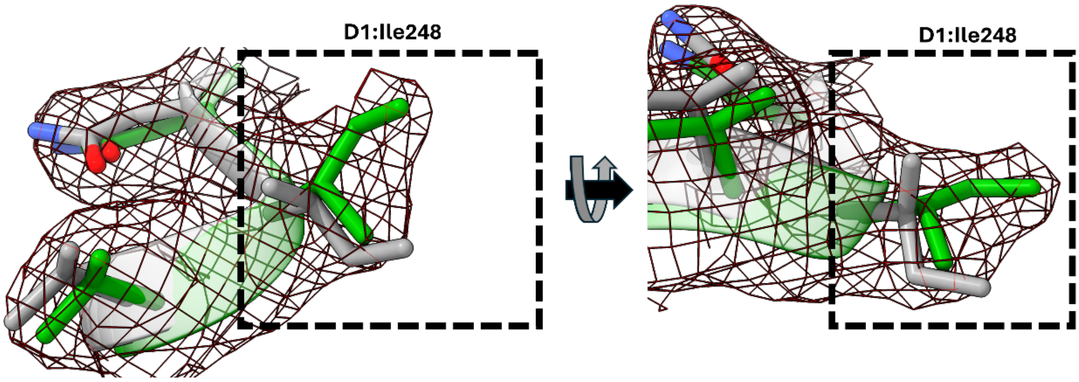

Fig. S7: The D1:Ile248 residue in the current Arabidopsis model (green) with the neighbouring residue on each side aligned with the same residues from the *T. vestitus* model (grey) (9EVX). The Coulomb potential map from the unsharpened map at a contour level of 2.11 RMSD is shown as a mesh. A black box indicates the residue of interest (D1:Ile248). The two images show different angles of the same map/model.

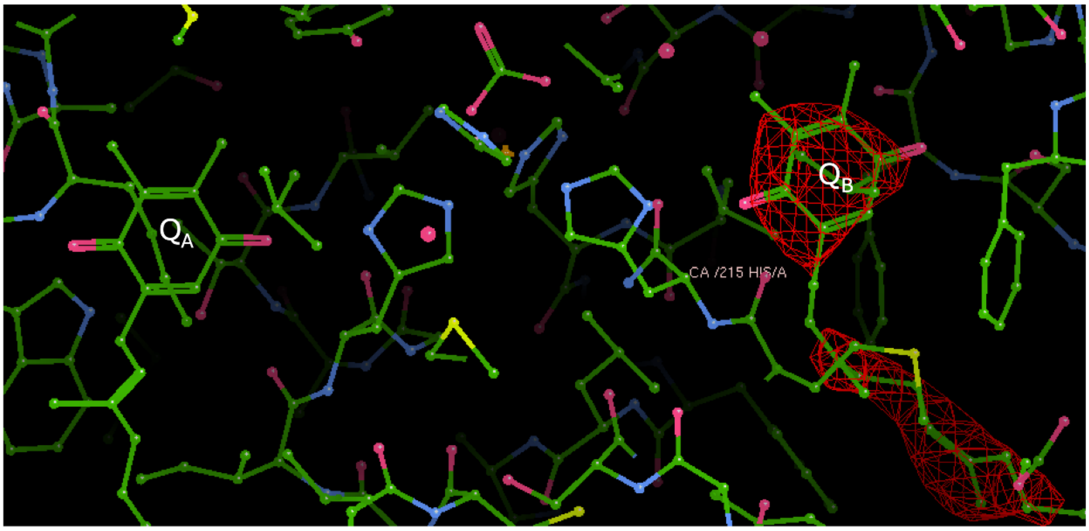

Fig. S8: Difference map analysis at the Q_B site. The B-factor of Q_B was adjusted to match the average B-factor of Q_A, and a difference map was generated using Servalcat (Yamashita et al., 2021). The F_oF_c map reveals a strong negative difference in density (shown in red mesh) at the Q_B site, suggesting reduced occupancy or higher mobility of Q_B in our Arabidopsis PS II structure. The sigma value of the map is 32.

Yamashita K, Palmer CM, Burnley T, Murshudov GN. Cryo-EM single-particle structure refinement and map calculation using Servalcat. (2021) *Acta Crystallogr D Struct Biol*. 77(10),1282-1291.

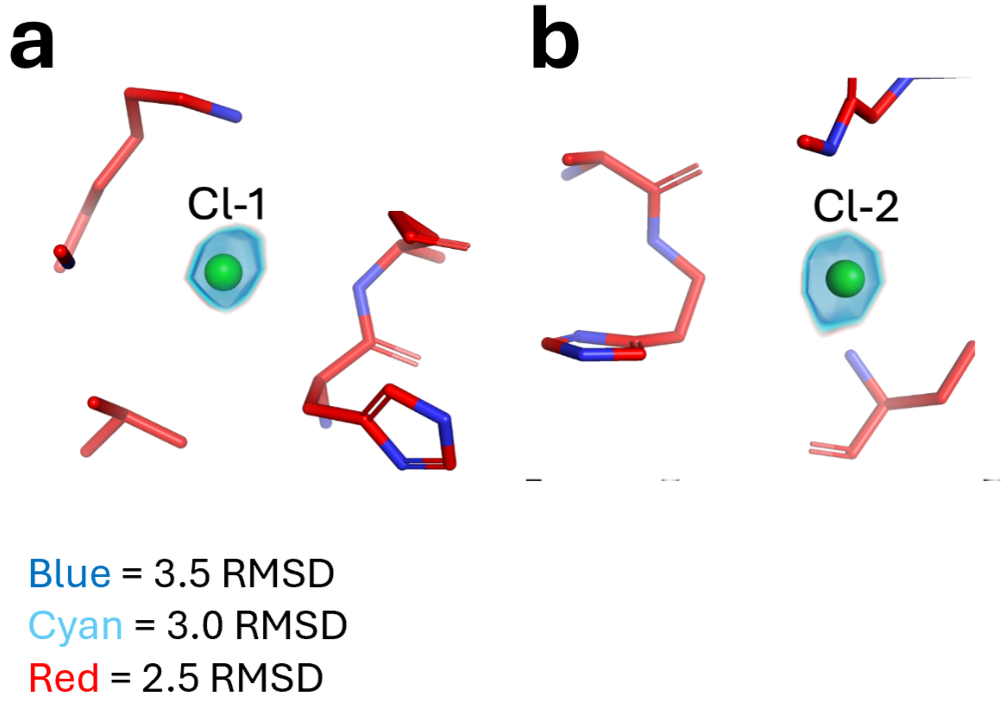

Fig. S9: Coulomb potential density around the Cl1 **(a)** and Cl2 **(b)** Chloride ions. Chloride ions are shown as green balls, with nearby residues as red sticks. The Coulomb potential from the Locally sharpened map is shown at contour levels of 3.5 RMSD (blue), 3.0 RMSD (cyan) and 2.5 RMSD (red).

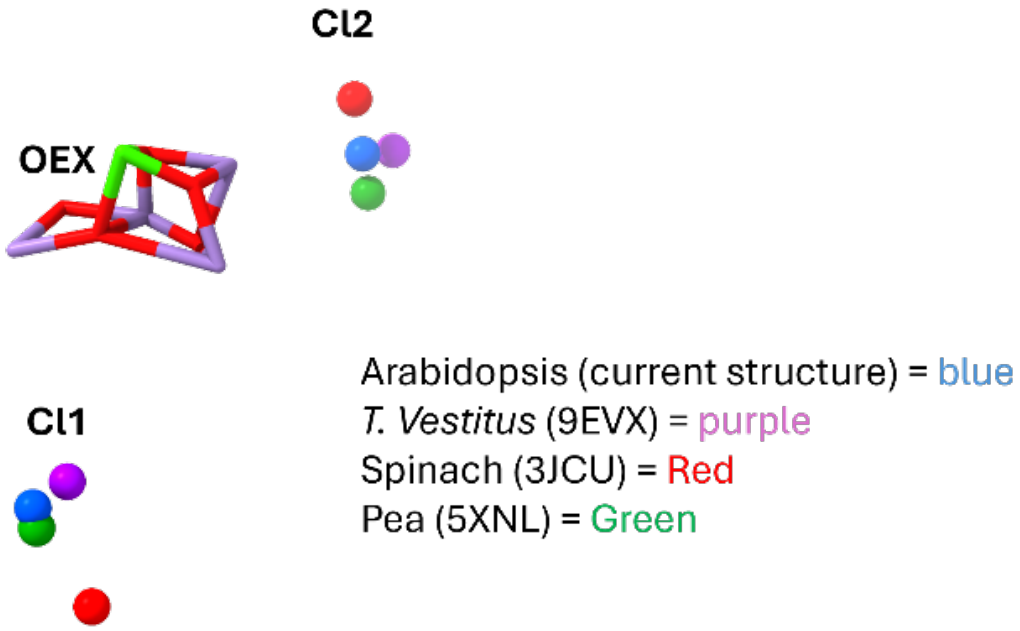

Fig. S10: Comparison of the Cl^- ion positions in the Arabidopsis (blue), *T. vestitus* (9EVX, purple), Spinach (3JCU, red) and Pea (5XNL, green) structures. Cl^- ions are shown as coloured spheres. The Mn-cluster of from the Arabidopsis structure is shown as a collection of purple, red and green sticks.

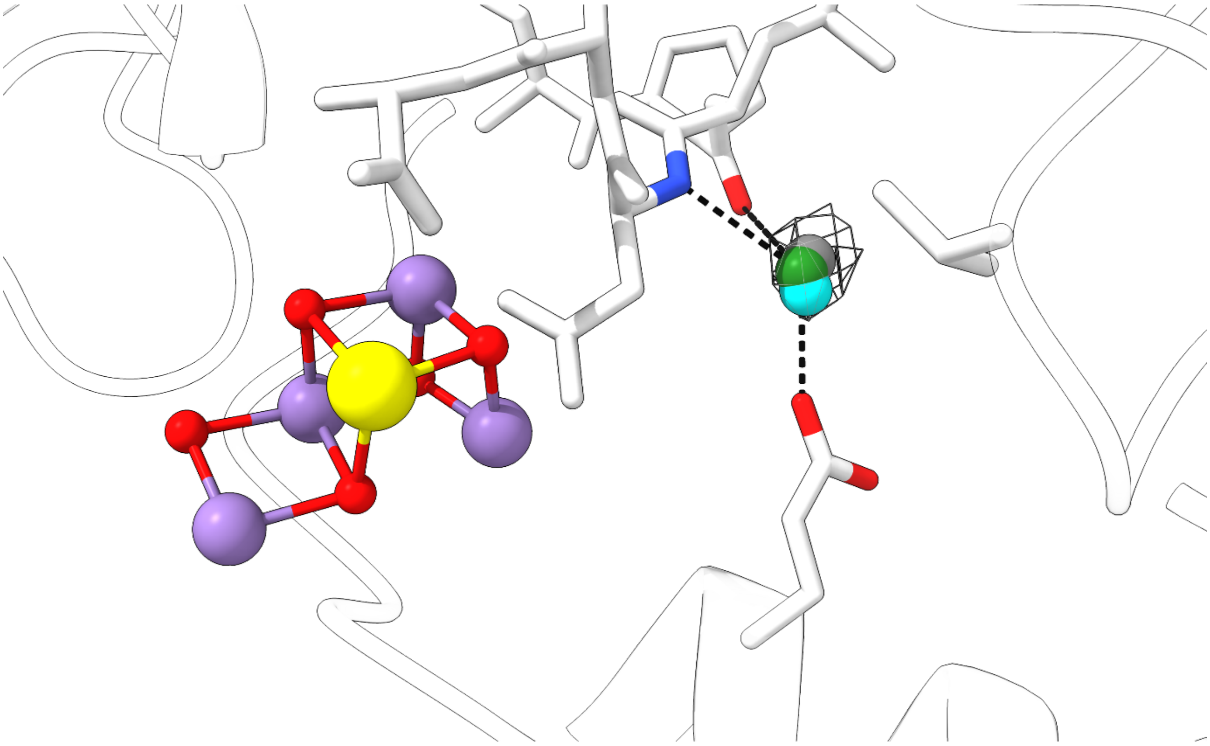

Fig. S11: Potential hydrogen bonding interactions between the water modelled at the bottleneck region of the O1 channel and the surrounding protein structure. A water in this position is found in Arabidopsis (water A:539 current structure, green sphere), *T. vestitus* (9EVX, grey sphere) and Pea (5XNL, cyan sphere). Potential hydrogen bonds are shown as black dashed lines, between the water and the D1:E329, D1:P340 and D1:D342 residues, corresponding to distances of 2.7 Å, 3.5 Å and 3.0 Å, respectively. The mesh around the bottleneck water represents the Coulomb density from the locally sharpened Coulomb potential map at a contour level of 1.29 RMSD.

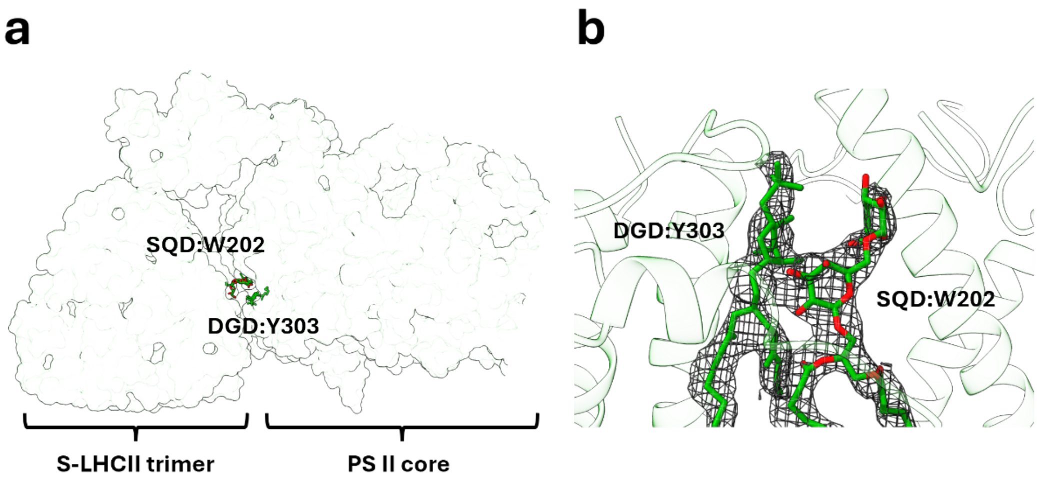

Fig. S12: Digalactosyl diacylglycerol (DGD) and sulfoquinovosyl diacylglycerol (SQD) lipids found at the interface between the PS II core and the LHC II trimer. **(a)** Location of the SQD:W202 and DGD:Y303 lipids within the PS II structure. The surface of the PS II core and strongly bound LHC II trimer is shown in faint green. The newly modelled SQD and DGD lipids are shown as sticks. **(b)** The fitting of the lipids within the locally sharpened Coulomb potential map at a contour level of 1.29 RMSD. The map is shown as a black mesh. The lipids are shown in green with red sections indicating oxygen atoms.

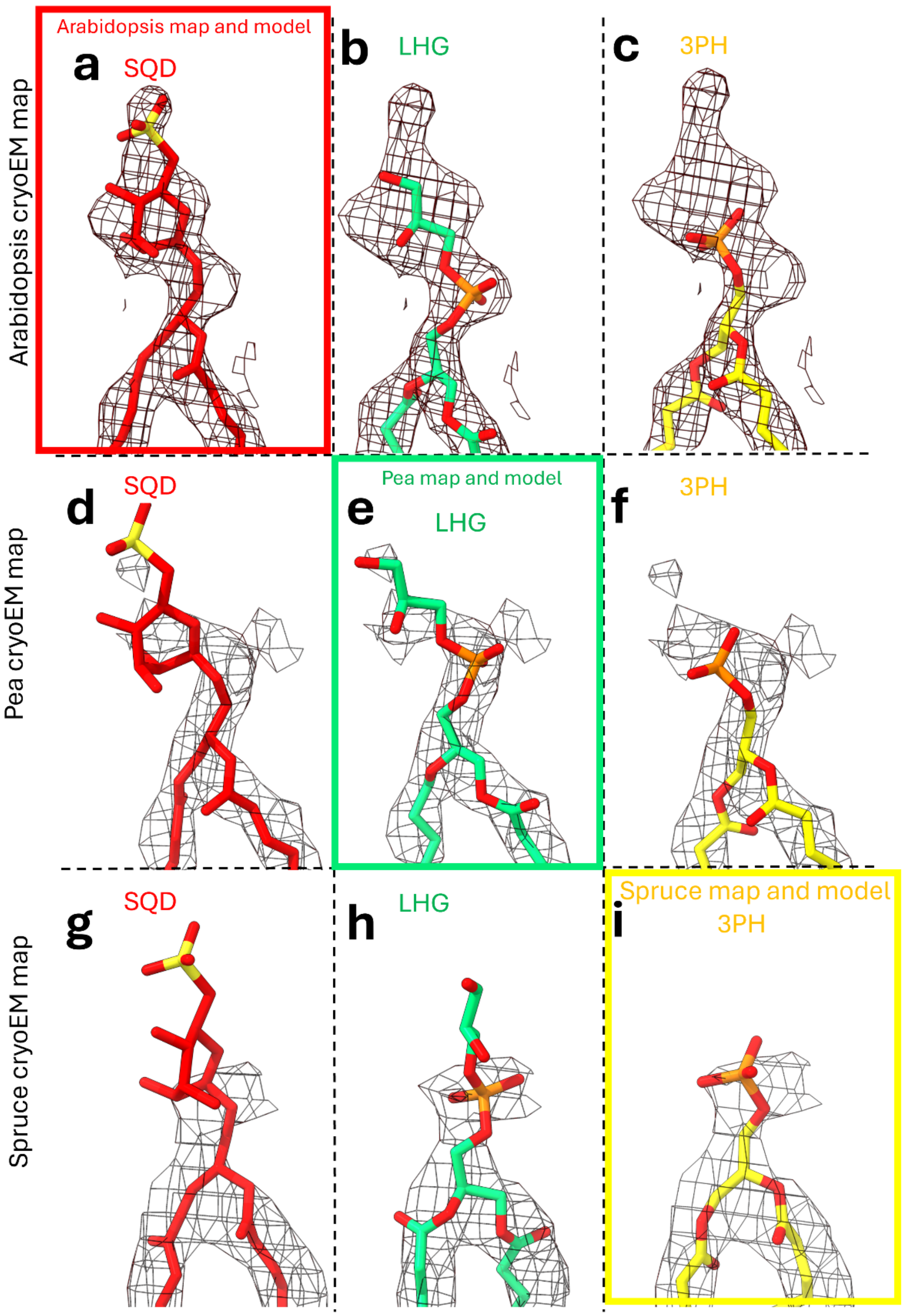

Fig. S13: Fitting ligands into the first Coulomb potential density at the s-LHCII:PS II core interface. The Coulomb potential density is shown from the Arabidopsis map **(a-c)** (full map at 1.62 RMSD), the pea map **(d-f)** (emd_6741) and the spruce map **(g-i)** (emd_16389). Into this density blob is fit, the ligand from the Arabidopsis model sulfoquinovosyl diacylglycerol (SQD) **(a,d,g)**, the ligand from the pea model (5XNL) dipalmitoyl-phosphatidyl-glycerol (LHG) **(b,e,h)**, and the ligand from the spruce model (8C29) Phosphatidic acid (3PH) **(c,f,i)**. Coloured boxes indicate when the map and model from the same organism are used. The fits show that the ligands from other structures do not fit well into the maps from other organisms, suggesting that the lipids at this position differ between organisms.

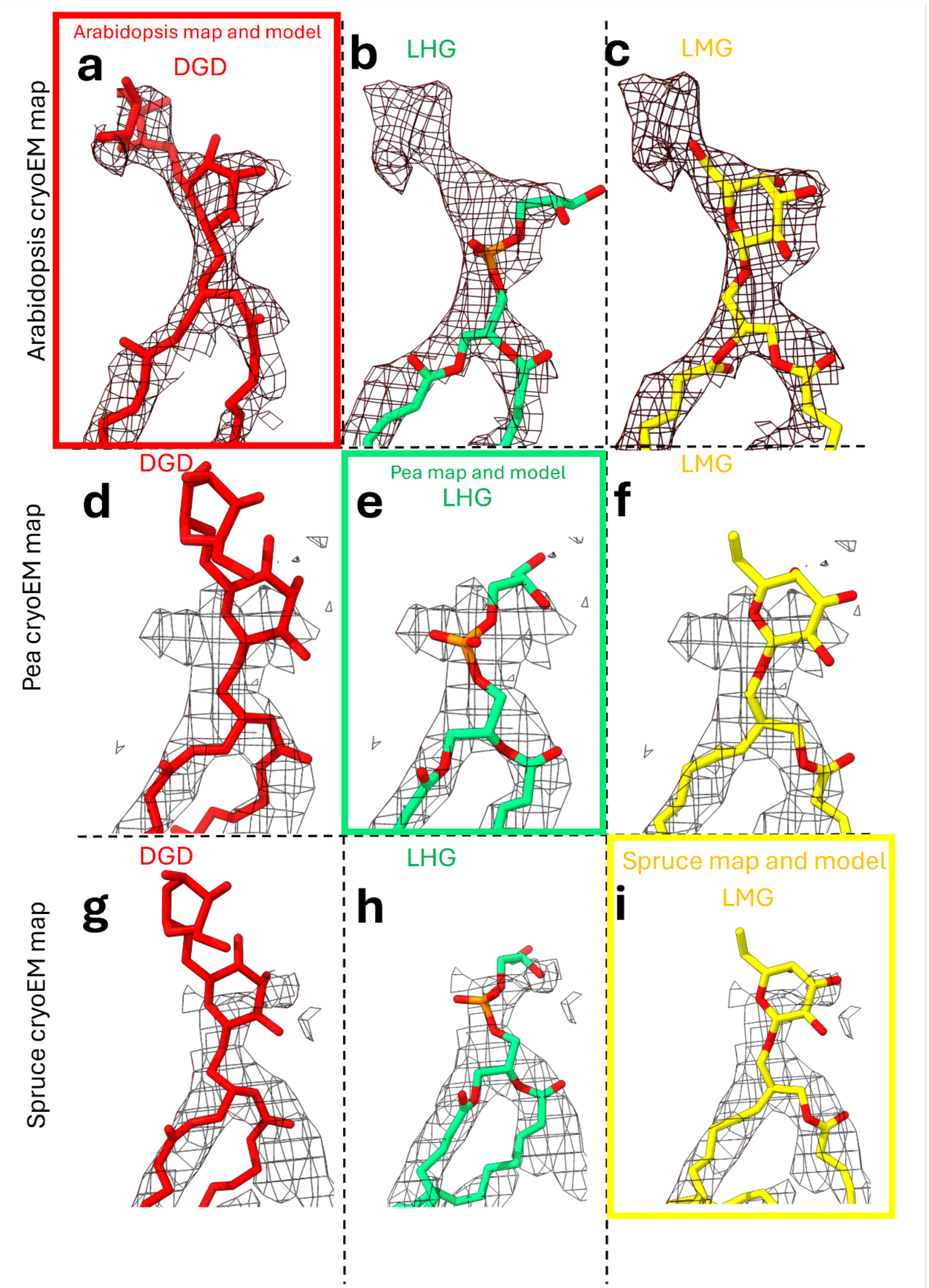

Fig. S14: Fitting ligands into the second Coulomb potential density at the s-LHCII:PS II core interface. The Coulomb potential density is shown from the Arabidopsis map **(a-c)** (full map at 1.62 RMSD), the pea map **(d-f)** (emd_6741) and the spruce map **(g-i)** (emd_16389). Into this density is fit, the ligand from the Arabidopsis model digalactosyl diacylglycerol (DGD) **(a,d,g)**, the ligand from the pea model (5XNL) dipalmitoyl-phosphatidyl-glycerol (LHG) **(b,e,h)**, and the ligand from the spruce model (8C29) monogalactosyl-diglyceride (LMG) **(c,f,i)**. Coloured boxes indicate when the map and model from the same organism are used. The fits show that the ligands from other structures do not fit well into the maps from other organisms, suggesting that the ligand at this position differs between organisms.

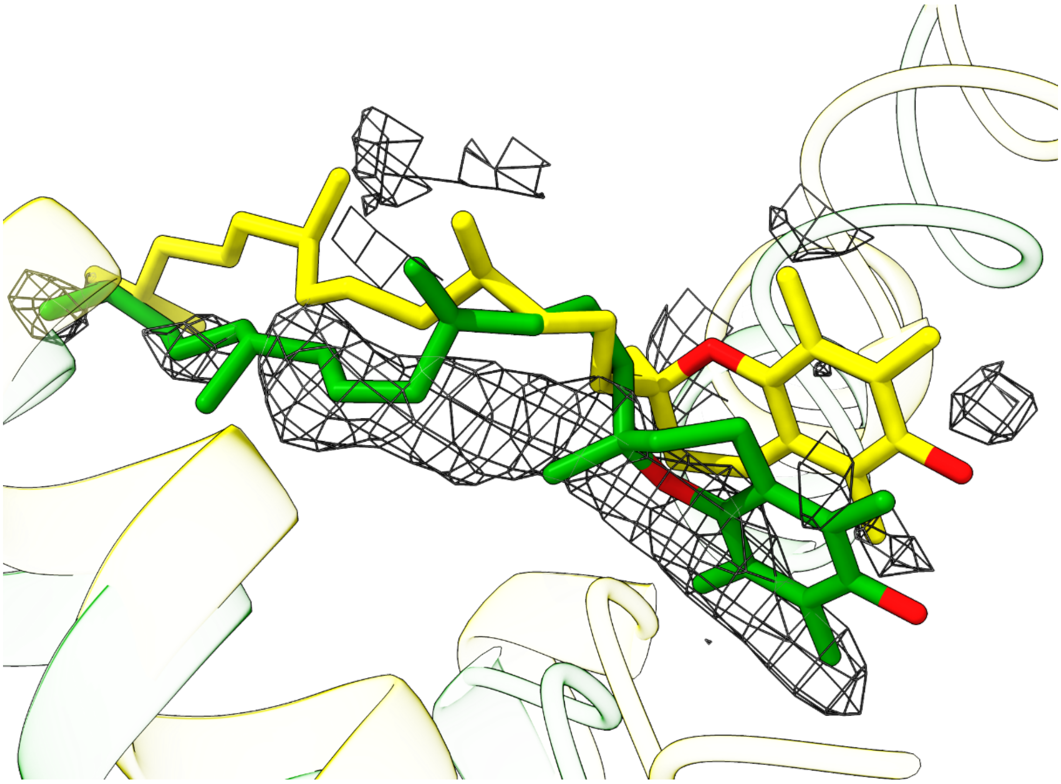

Fig. S15: Fitting of an α-tocopherol molecule. The putative α-tocopherol molecule which is not included in the final Arabidopsis model is shown in green, with red sections indicating oxygen atoms. The position of the α-tocopherol modelled in the spruce PS II structure (8C29) is shown in yellow. The locally sharpened Coulomb potential map at a contour level of 1.29 RMSD is shown as a black mesh. The surrounding protein is shown as faint ribbons for Arabidopsis (green) and spruce (yellow).

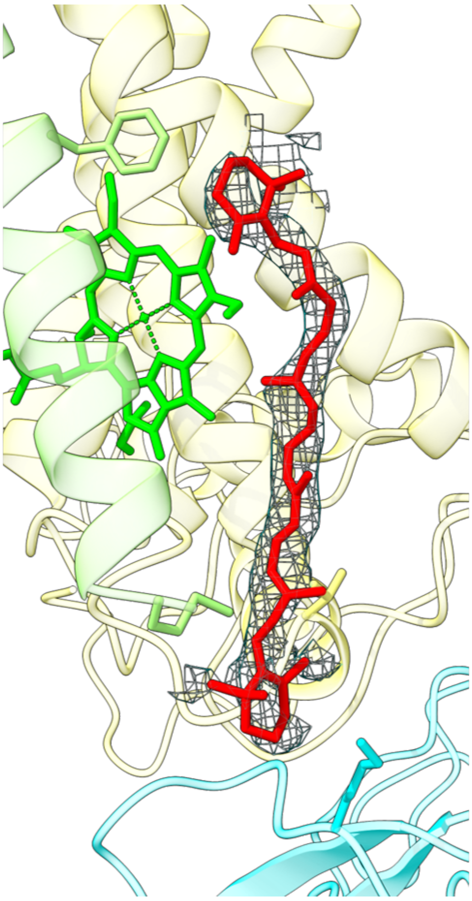

Fig. S16: The environment and Coulomb density of a potential β-carotene is shown for one of the monomers. The modelled β-carotene is shown in red. The electrostatic density from the local sharpened Coulomb potential map within 1.5 Å of the β-carotene is shown at a contour level of 1.38 RMSD. The map is shown as a mesh to demonstrate the accuracy of the fitting of the potential new β-carotene. The nearest subunits to the potential β-carotene molecule are D1 (yellow), PsbI (green) and PsbO (cyan). The nearest chlorophyll molecule (Chla:A405) is shown in lime green. The closest interactions between the potential β-carotene and the surrounding proteins are: 4.1 Å to D1:Val102, 3.9 Å to PsbI:Phe15, 3.2 Å to PsbI:Met1, 3.9 Å to PsbO:Lys160, 5.2 Å to Chla:A410.

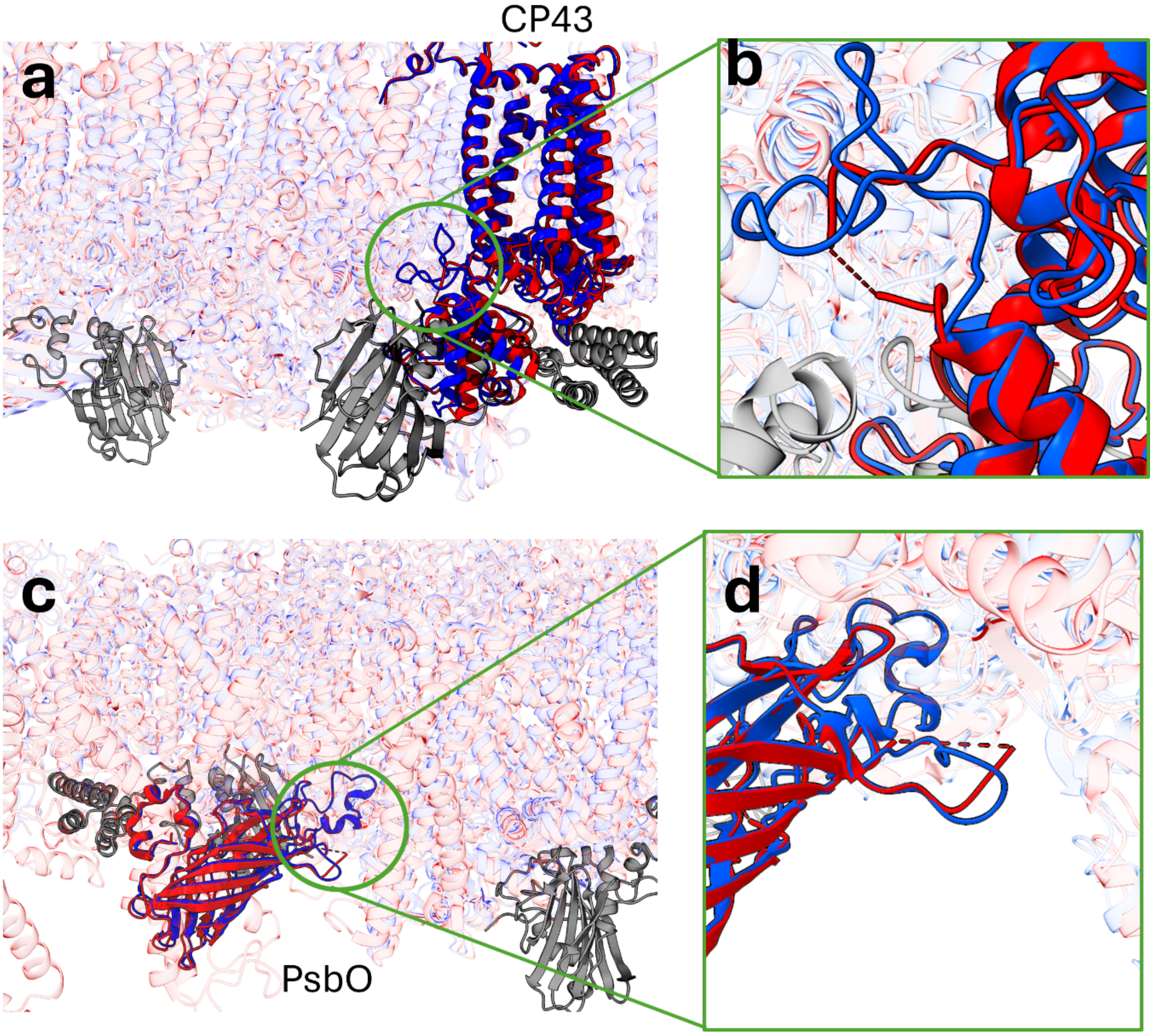

Fig. S17: Comparison of the current Arabidopsis structure with the previous structure which lacks the the PsbP and PsbQ subunits (7OUI). The current Arabidopsis structure is shown in transparent blue, older Arabidopsis structure is shown in transparent red. Subunits which show differences on the donor side are shown in bold, CP43 (**a,b**) and PsbO (**c,d**). The PsbP and PsbQ subunits which are absent in the 7OUI structure are shown in black. Significant differences between CP43 and PsbO subunits in the two structures are circled in green, and expanded in panels **c** and **d**

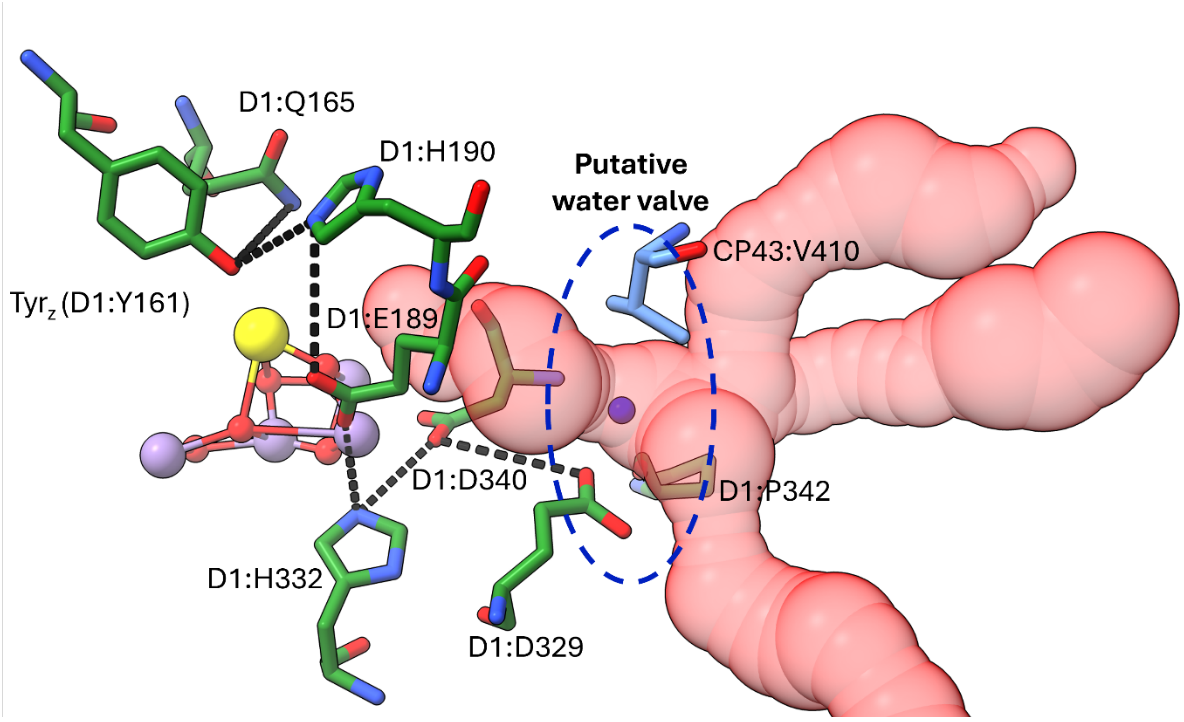

Fig. S18: Potential connections from the Y_z, D1:165, D1:190 triad (Li et al., 2024) and the putative water valve. Potential hydrogen bonds are shown in black. Key residues from D1 (green) and CP43 (light blue) are shown as sticks, with red and blue sections indicating oxygen and nitrogen atoms, respectively. The O1 channel is shown in red and the Mn-cluster is shown as a collection of purple, red and yellow spheres.

Supplementary Table 1. Atom inclusion and Q-scores for selected subunits in the Arabidopsis (current structure), Pea (5XNL) and Spinach (3JCU) structures. The Atom and Q-scores were calculated at the contour level recommended in the validation reports. The Atom inclusion and Q-scores are only shown for one monomer.

Organism	Subunit	Atom inclusion (%)	Q-score
Arabidopsis	PsbA	99.5%	0.669
	PsbP	95.3%	0.639
	PsbQ	78.9%	0.587
Pea (5XNL)	PsbA	87.3%	0.643
	PsbP	62.4%	0.576
	PsbQ	7.6%	0.409
Spinach (3JCU)	PsbA	62.3%	0.531
	PsbP	0.2%	0.396
	PsbQ	0.4%	0.337

Supplementary Table 2: Statistics of cryo-EM data and structural analysis of the C2S2-type PSII supercomplex refined at 2.44 Å resolution.

**Data collection and processing**	
Hardware	
Microscope	Titan Krios
Detector (mode)	FEI Falcon IV
Voltage (keV)	300
Spherical aberration	2.7
Magnification	165,000 x
Electron exposure (e/Å^2)	40
Defocus range (μm)	-0.8 to -2.0 (0.2 increments)
Pixel size (Å)	0.7155
Symmetry imposed	C2
Initial particle images (no.)	3,054,062
Final particle images (no.)	72 301
Map resolution (Å)	2.44 (C_2S_2 - masked)
FSC threshold	0.143
**Refinement**	
Initial models used (PDB code)	3JCU
Model composition	
Non-hydrogen atoms	76906
Protein residues	7548
Ligands	334
Water molecules	1206
B factors (Å^2)	
Protein (mean)	61.92
Ligand (mean)	65.11
R.m.s. deviations	
Bond lengths (Å)	0.008
Bond angles (°)	0.726
Validation	
MolProbity score	1.76
Clash score	10.62
Rotamer outliers (%)	1.37
Ramachandran plot	
Favored (%)	97.42
Allowed (%)	2.55
Outliers (%)	0.03

Supplemental table 3: Details of the individual Photosystem II subunits. The protein name is shown in bold, while the gene name is contained within parenthesis. The accession code used to identify the protein in UniProt is shown before the sequence. The sequences have amino acids letters colored in red and black: **black** letters, all the amino acids that were modelled, **red**, amino acids that are not observed in a 3D map of Arabidopsis thaliana. Each subunit is shown using red sticks and fit into the local sharpened electron density map (transparent blue surface) at a contour level of 1.5 RMSD and a distance of 2. The location of each subunit within the Photosystem II dimer is also shown, with the subunit highlighted in red and the whole dimer shown in transparent grey.

Chain ID	Protein Name (Gene name)	MW (kDa)	Sequence (mature protein)	Model fit into the electrostatic potential map	Location of subunit within the PS II dimer
A,a	**D1** (PsbA)	37.98	>P83755<br>MTAILERRESESLWGRFCNWITSTENRLYIGWF<br>GVLMIPTLLTATSVFIIAFIAAPPVDIDGIREPVS<br>GSLLYGNNIISGAIIPTSAAIGLHFYPIWEAASVD<br>EWLYNGGPYELIVLHFLLGVACYMGREWELSF<br>RLGMRPWIAVAYSAPVAAATAVFLIYPIGQGSF<br>SDGMPLGISGTFNFMIVFQAEHNILMHPFHM<br>LGVAGVFGGSLFSAMHGSLVTSSLIRETTENES<br>ANEGYRFGQEEETYNIVAAHGYFGRLIFQYASF<br>NNSRSLHFFLAAWPVVGIWFTALGISTMAFNL<br>NGFNFNQSVVDSQGRVINTWADIINRANLGM<br>EVMHERNAHNFPLDLAA	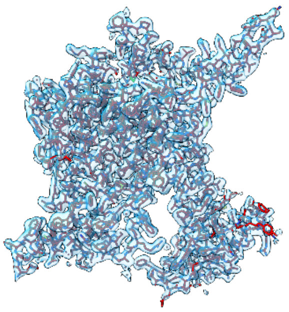	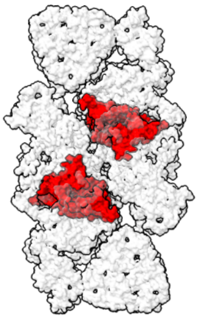
B,b	**CP47** (PsbB)	56.04	>P56777<br>MGLPWYRVHTVVLNDPGRLLAVHIMHTALVA<br>GWAGSMALYELAVFDPSDPVLDPMWRQGM<br>FVIPFMTRLGITNSWGGWNITGGTITNPGLWS<br>YEGVAGAHIVFSGLCFLAAIWHWVYWDLEIFC<br>DERTGKPSLDLPKIFGIHLFLSGVACFGFGAFHV<br>TGLYGPGIWVSDPYGLTGKVQPVNPAWGVEG<br>FDPFVPGGIASHHIAAGTLGILAGLFHLSVRPPQ<br>RLYKGLRMGNIETVLSSSIAAVFFAAFVVAGTM<br>WYGSATTPIELFGPTRYQWDQGYFQQEIYRRV<br>SAGLAENQSLSEAWAKIPEKLAFYDYIGNNPAK<br>GGLFRAGSMDNGDGIAVGWLGHPVFRNKEG<br>RELFVRRMPTFFETFPVVLVDGDGIVRADVPFR<br>RAESKYSVEQVGVTVEFYGGELNGVSYSDPAT<br>VKKYARRAQLGEIFELDRATLKSDGVFRSSPRG<br>WFTFGHASFALLFFFGHIWHGARTLFRDVFAGI<br>DPDLDAQVEFGAFQKLGDPTTKRQAV	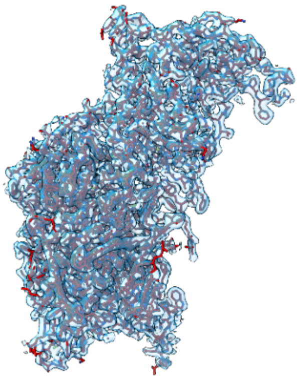	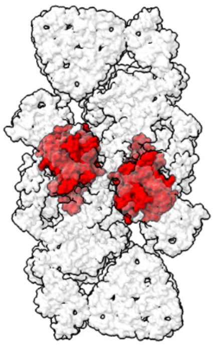
C,c	**CP43** (PsbC)	50.04	>P56778<br>TLFNGTLALAGRDQETTGFAWWAGNARLINLS<br>GKLLGAHVAHAGLIVFWAGAMNLFEVAHFVP<br>EKPMYEQGLILLPHLATLGWGVGPGGEVIDTF<br>PYFVSGVLHLISSAVLGFGGIYHALLGPETLEESF<br>PFFGYVVWKDRNKMTTILGIHLILLGVGAFLLVFK<br>ALYFGGVYDTWAPGGGDVRKITNLTLSPSVIFG<br>YLLKSPFGGEGWIVSVDDLEDIIGGHVWLGSICI<br>FGGIWHILTKPFAWARRALVWSGEAYLSYSLA<br>ALSVCGFIACCFVWFNNTAYPSEFYGPTGPEAS<br>QAQAFTFLVRDQRLGANVGSAQGPTGLGKYL<br>MRSPTGEVIFGGETMRFWDLRAPWLEPLRGP<br>NGLDLSRLKKDIQPWQERRSAEYMTHAPLGSL<br>NSVGGVATEINAVNYVSPRSWLSTSHFVLGFFL<br>FVGHLWHAGRARAAAAGFEKGIDRDFEPVLS<br>MTPLN	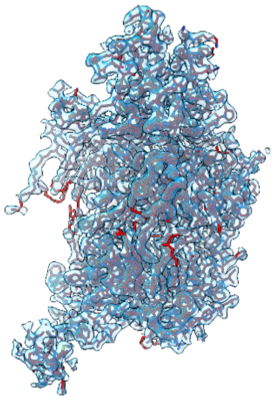	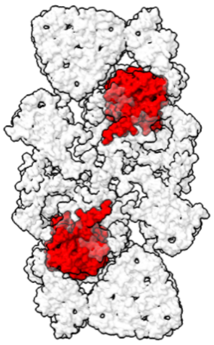

D,d	**D2**<br><br>(PsbD)	39.42	>P56761<br>TIALGKFTKDEKDLFDIMDDWLRRDRFVFVGW<br>SGLLLFPCAYFALGGWFTGTTFVTSWYTHGLAS<br>SYLEGCNFLTAAVSTPANSLAHSLLLLWGPEAQ<br>GDFTRWCQLGGLWAFVALHGAFALIGFMLRQ<br>FELARSVQLRPYNAIAFSGPIAVFVSVFLIYPLGQ<br>SGWFFAPSFGVAAIFRFILFFQGFHNWTLNPFH<br>MMGVAGVLGAALLCAIHGATVENTLFEDGDG<br>ANTFRAFNPTQAEETYSMVTANRFWSQIFGV<br>AFSNKRWLHFFMLFVPVTGLWMSALGVVGLA<br>LNLRAYDFVSQEIRAAEDPEFETFYTKNILLNEGI<br>RAWMAAQDQPHENLIFPEEVLPRGNAL	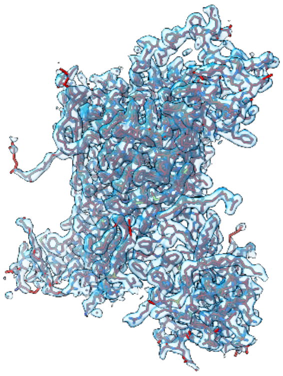	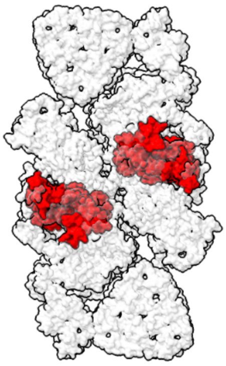
E,e	**Cytochrome b559α**<br>(PsbE)	9.39	>P56779<br>MSGSTGERSFADIITSIRYWVIHSITIPSLFIAGW<br>LFVSTGLAYDVFGSPRPNEYFTESRQGIPLITGR<br>FDSLEQLDEFSRSF	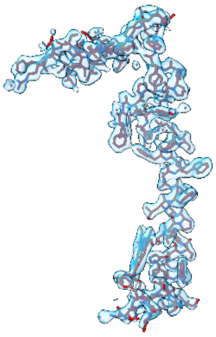	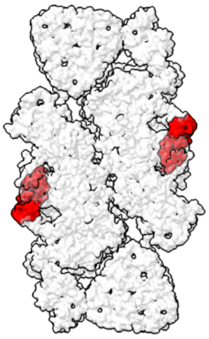
F,f	**Cytochrome b559β**<br>(PsbF)	4.42	>P62095<br>MTIDRTYPIFTVRWLAVHGLAVPTVFFLGSISA<br>MQFIQR	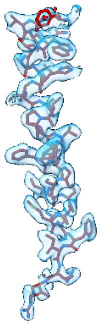	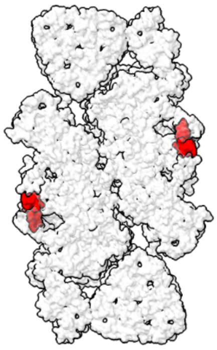
H,h	**Photosystem II reaction center protein H**<br>(PsbH)	7.57	>P56780<br>ATQTVEDSSRSGPRSTTVGKLLKPLNSEYGKVA<br>PGWGTTPLMGVAMALFAVFLSIILEIYNSSVLL<br>DGISVN	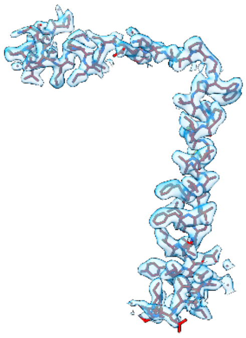	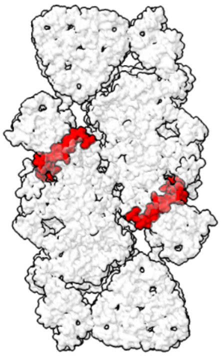

I,i	**Photosystem II reaction center protein I** (PsbI)	4.17	>P62100 MLTLKLFVYTVVIFFVSLFIFGFLSNDPGRNPGREE	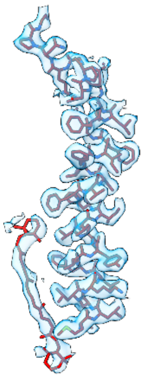	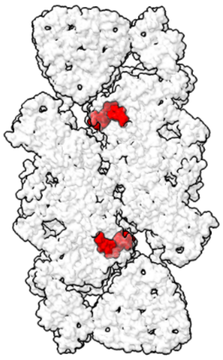
J,j	**Photosystem II reaction center protein J** (PsbJ)	4.12	>P56781 MADTTGRIPLWVIGTVAGILVIGLIGIFFYGSYSGLGSSL	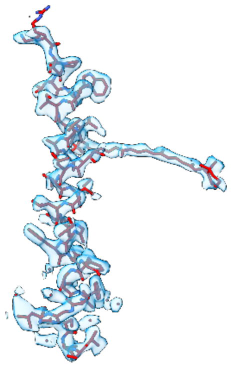	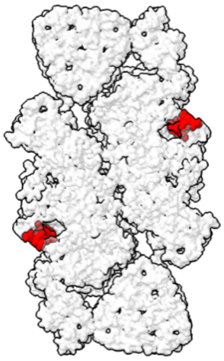
K,k	**Photosystem II reaction center protein K** (PsbK)	4.24	>P56782 KLPEAYAFLNPIVDVMPVIPLFFLLLAFVWQAAVSFR	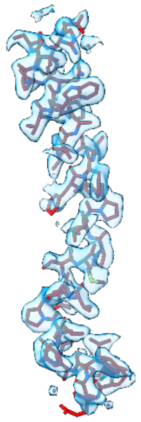	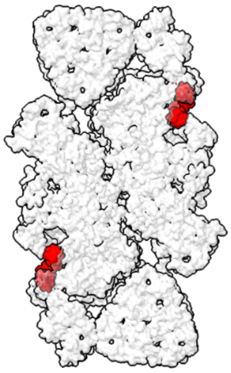
L,l	**Photosystem II reaction center protein L** (PsbL)	4.47	>P60129 MTQSNPNEQSVELNRTSLYWGLLLIFVLAVLFSNYFFN	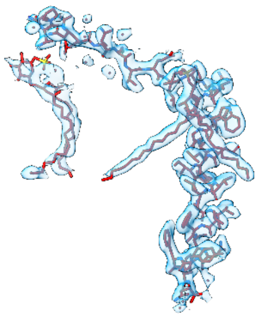	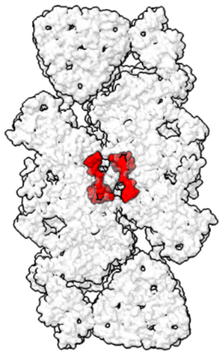

M,m	**Photosystem II reaction center protein M** (PsbM)	3.78	>P62109<br>MEVNILAFIATALFILVPTAFLLIIYVKTVSQND	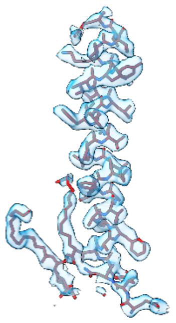	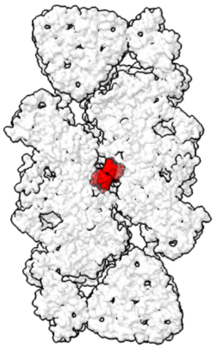
O,o	**Oxygen-evolving enhancer protein 1-1** (PsbO1)	26.57	>P23321<br>EGAPKRLTYDEIQSKTYMEVKGTGTANQCPTID<br>GGSETFSFKPGKYAGKKFCFEPTSFTVKADSVSK<br>NAPPEFQNTKLMTRLTYTLDEIEGPFEVASDGS<br>VNFKEEDGIDYAAVTVQLPGGERVPFLFTVKQL<br>DASGKPDSFTGKFLVPSYRGSSFLDPKGRGGST<br>GYDNAVALPAGGRGDEEELVKENVKNTAASV<br>GEITLKVTKSKPETGEVIGVFESLQPSDTDLGAK<br>VPKDVKIQGVWYGQLE	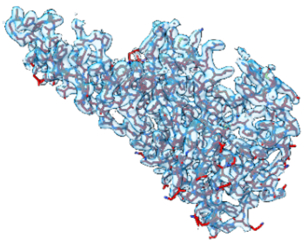	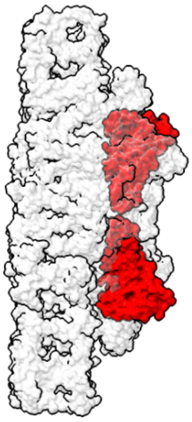
P,p	**Oxygen-evolving enhancer protein 2-1** (PsbP1)	28.10	>Q42029<br>MAYSACFLHQSALASSAARSSSSSSQRHVSLS<br>KPVQIICKAQQSHEDDNSAVSRRLALTLLVGAA<br>AVGSKVSPADAAYGEAANVFGKPKTNTDFLPY<br>NGDGFKVQVPAKWNPSKEIEYPGQVLRFEDN<br>FDATSNLNVMVTPTDKKSITDYGSPEEFLSQVN<br>YLLGKQAYFGETASEGGFDNNAVATANILESSS<br>QEVGGKPYYYLSVLTRTADGDEGGKHQLITATV<br>NGGKLYICKAQAGDKRWFKGARKFVESAATSF<br>SVA	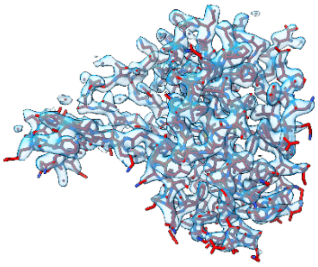	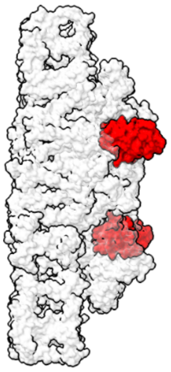
Q,q	**Oxygen-evolving enhancer protein 3-1** (PsbQ1)	23.87	>Q9XFT3<br>MASMGGLHGASPAVLEGSLKINGSSRLNGSGR<br>VAVAQRSRLVVRAQQSEETSRRSVIGLVAAGL<br>AGGSFVQAVLADAISIKVGPPPAPSGGLPAGTD<br>NSDQARDFALALKDRFYLQPLPPTEAAARAKES<br>AKDIINVKPLIDRKAWPYVQNDLRSKASYLRYD<br>LNTIISSKPKDEKKSLKDLTTKLFDTIDNLDYAAK<br>KKSPSQAEKYYAETVSALNEVLAKLG	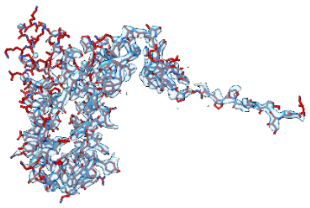	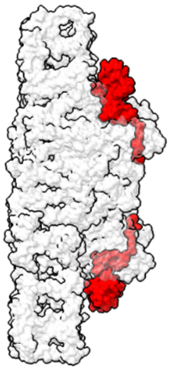

T,t	**Photosystem II reaction center protein Tc** (PsbTc)	3.82	>P61839<br>MEALVYTFLLVSTLGIIFFAIFFREPPKISTKK	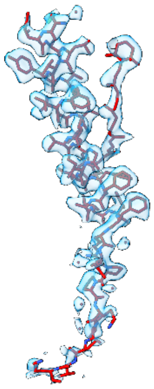	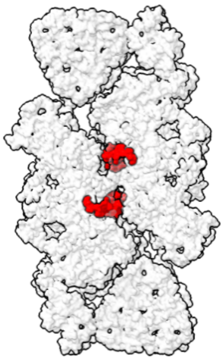
U,u	**Photosystem II reaction center protein Tn** (PsbTn)	3.17	>Q39195<br>EPKRGTEAAKKKYAQVCVTMPTAKICRY	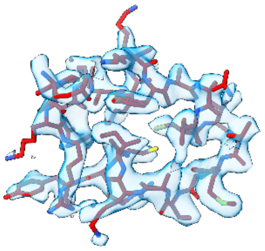	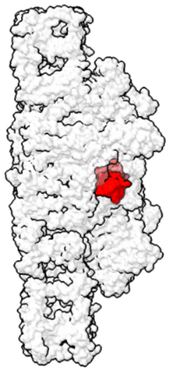
W,w	**Photosystem II reaction center W protein** (PsbW)	6.04	>Q39194<br>LVDERMSTEGTGLPFGLSNNLLGWILFGVFGLI WTFFFVYTSSLEEDEESGLSL	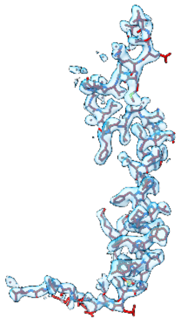	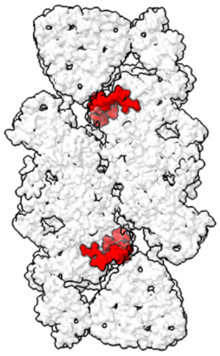
X,x	**Photosystem II reaction center protein X** (PSII-X)	4.18	>Q9SKI3<br>AGSGISPSLKNFLLSIASGGLVLTVIIGVVVGVSN FDPVKRT	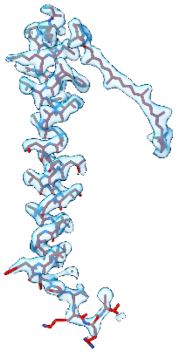	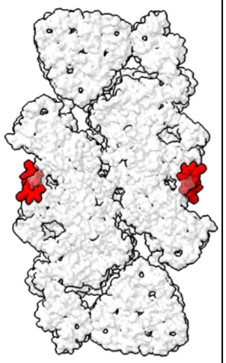

Z,z	**Photosystem II reaction center protein Z** (PsbZ)	6.57	>P56790<br>MTIAFQLAVFALIITSSILLISVPVVFASPDGWSS NKNVVFSGTSLWIGLVFLVGILNSLIS	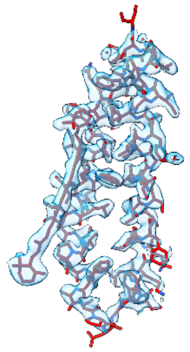	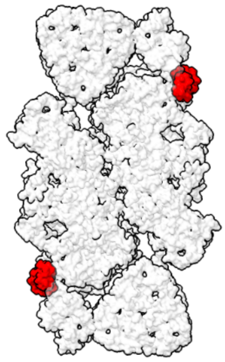
G,g	**Chlorophyll a-b binding protein 1** (LHCB1.3)	24.86	>P04778<br>RKTVAKPKGPSGSPWYGSDRVKYLGPFSGESP SYLTGEFPGDYGWDTAGLSADPETFARNRELE VIHSRWAMLGALGCVFPELLARNGVKFGEAV WFKAGSQIFSDGGLDYLGNPSLVHAQSILAIW ATQVILMGAVEGYRVAGNGPLGEAEDLLYPGG SFDPLGLATDPEAFAELKVKELKNGRLAMFSMF GFFVQAIVTGKGPIENLADHLADPVNNNAWA WAFATN	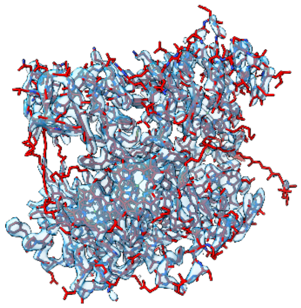	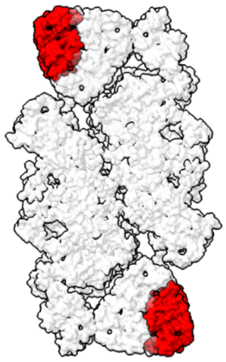
N,n	**Chlorophyll a-b binding protein 1** (LHCB1.3)	24.86	>P04778<br>RKTVAKPKGPSGSPWYGSDRVKYLGPFSGESP SYLTGEFPGDYGWDTAGLSADPETFARNRELE VIHSRWAMLGALGCVFPELLARNGVKFGEAV WFKAGSQIFSDGGLDYLGNPSLVHAQSILAIW ATQVILMGAVEGYRVAGNGPLGEAEDLLYPGG SFDPLGLATDPEAFAELKVKELKNGRLAMFSMF GFFVQAIVTGKGPIENLADHLADPVNNNAWA WAFATN	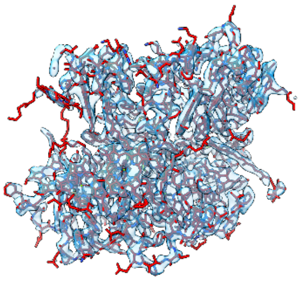	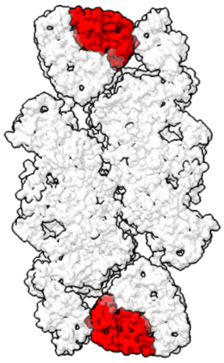
Y,y	**Chlorophyll a-b binding protein 1** (LHCB2.2)	24.86	>Q9S7J7<br>MATSAIQQSSFAGQTALKPSSDLIQKVGVLGG GRVTMRRTVKSTPQSIWYGPDRPKYLGPFSEN TPSYLTGEYPGDYGWDTAGLSADPETFAKNRE LEVIHSRWAMLGALGCTFPEILSKNGVKFGEAV WFKAGSQIFSEGGLDYLGNPNLIHAQSILAIWA VQVVLMGFIEGYRIGGGPLGEGLDPLYPGGAF DPLNLAEDPEAFSELKVKELKNGRLAMFSMFG FFVQAIVTGKGPIENLFDHLADPVANNAWSYA TNFVPGK	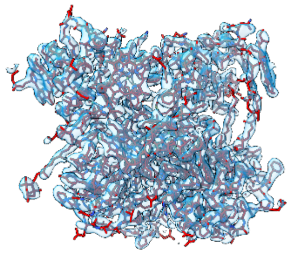	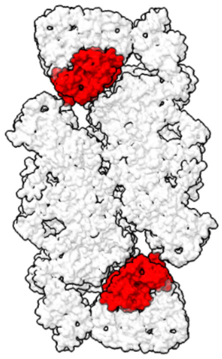

R,r	**CP29** (LHCB4.1)	28.16	>Q07473<br><br>AAPKKSAKKTVTTDRPLWYPGAISPDWLDGSL VGDYGFDPFGLGKPAEYLQFDIDSLDQNLAKN LAGDVIGTRTEAADAKSTPFQPYSEVFGIQRFR ECELIHGRWAMLATLGALSVEWLTGVTWQDA GKVELVDGSSYLGQPLPFSISTLIWIEVLVIGYIEF QRNAELDSEKRLYPGGKFFDPLGLAADPEKTA QLQLAEIKHARLAMVAFLGFAVQAAATGKGPL NNWATHLSDPLHTTIIDTFSSS	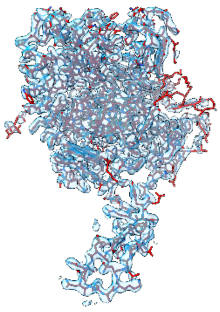	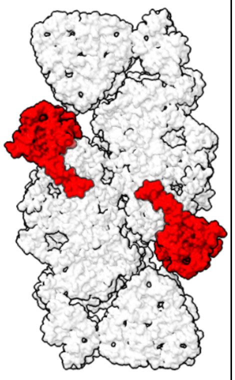
S,s	**CP26** (LHCB5)	25.21	>Q9XF89<br><br>SKAVSETSDELAKWYGPDRRIFLPDGLLDRSEIP EYLNGEVAGDYGYDPFGLGKKPENFAKYQAFE LIHARWAMLGAAGFIIPEALNKYGANCGPEAV WFKTGALLLDGNTLNYFGKNIPINLVLAVVAEV VLLGGAEYYRITNGLDFEDKLHPGGPFDPLGLA KDPEQGALLKVKEIKNGRLAMFAMLGFFIQAY VTGEGPVENLAKHLSDPFGNNLLTVIAGTAERA PTL	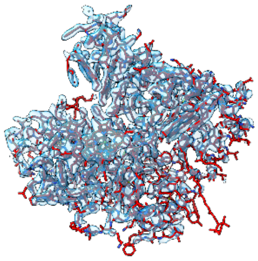	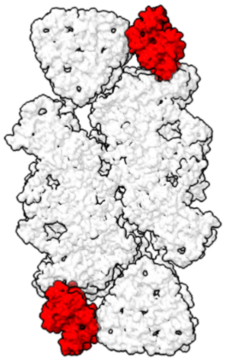

Supplemental table 4: Details of various ligands found within the structure.

Ligand Name	3-letter Code	# of ligands (full dimer)
β-CAROTENE	BCR	22
BICARBONATE ION	BCT	2
CHLOROPHYLL B	CHL	50
CHLORIDE ION	CL	4
CHLOROPHYLL A	CLA	156
DIGALACTOSYL DIACYL GLYCEROL (DGDG)	DGD	10
FE (II) ION	FE2	2
PROTOPORPHYRIN IX CONTAINING FE	HEM	2
WATER	HOH	1206
1,2-DIPALMITOYL-PHOSPHATIDYL-GLYCEROLE	LHG	18
1,2-DISTEAROYL-MONOGALACTOSYL-DIGLYCERIDE	LMG	10
DODECYL-BETA-D-MALTOSIDE	LMT	2
(3R,3'R,6S)-4,5-DIDEHYDRO-5,6-DIHYDRO-BETA,BETA-CAROTENE-3,3'-DIOL	LUT	18
(3S,5R,6R,3'S,5'R,6'S)-5',6'-EPOXY-6,7-DIDEHYDRO-5,6,5',6'-TETRAHYDRO-BETA,BETA-CAROTENE-3,5,3'-TRIOL; 9'-CIS-NEOXANTHIN	NEX	10
CA-MN4-O5 CLUSTER	OEX	2
PHEOPHYTIN A	PHO	4
2,3-DIMETHYL-5-(3,7,11,15,19,23,27,31,35-NONAMETHYL-2,6,10,14,18,22,26,30,34-HEXATRIACONTANONAENYL-2,5-CYCLOHEXADIENE-1,4-DIONE-2,3-DIMETHYL-5-SOLANESYL-1,4-BENZOQUINONE	PL9	4
1,2-DI-O-ACYL-3-O-[6-DEOXY-6-SULFO-ALPHA-D-GLUCOPYRANOSYL]-SN-GLYCEROL	SQD	8
(3S,5R,6S,3'S,5'R,6'S)-5,6,5',6'-DIEPOXY-5,6,5',6'-TETRAHYDRO-BETA,BETA-CAROTENE-3,3'-DIOL	XAT	8

Supplemental table 5: Ligands within individual subunits. The full name of the 3-letter codes for each ligand can be found in Supplemental table 4.

**Subunit**	**Ligand**	BCR	CHL	CL	CLA	DGD	HEM	LHG	LMG	LMT	LUT	NEX	SQD	XAT
D1		1		2	3								1	
CP47		3			16									
CP43		2			13	3			3					
D2		1			3	1		3	1	1			1	
PsbE							1							
PsbF														
PsbH														
PsbI														
PsbJ		1												
PsbK														
PsbL								1					1	
PsbM									1					
PsbO														
PsbP														
PsbQ														
PsbT		1												
PsbU														
PsbW													1	
PsbX		1												
PsbZ		1												
LHCB1.3			6		8			1			2	1		1
LHCB2.2			6		8	1		1			2	1		1
CP26			4		9			1			2	1		
CP29			3		10			1			1	1		1

Supplemental Table 6: Mn-cluster distances in this structure and other PS II structures

	Arabidopsis (this structure) 2.44 Å resolution	(Kern et al., 2018) XFEL 6DHE 2.05 Å resolution	Kato et al., 2021 High dose 1.95 Å resolution	Kato et al., 2021 Low dose 2.08 Å resolution
Mn1-Mn2	3.2	2.8	2.8	2.9
Mn1-Mn3	3.5	3.2	3.4	3.2
Mn1-Mn4	5.4	4.9	5.0	5.1
Mn2-Mn3	2.6	2.9	3.1	2.9
Mn3-Mn4	3.6	2.7	3.0	3.0
Mn1-Ca	3.8	3.4	3.5	3.6
Mn2-Ca	3.7	3.4	3.5	3.4
Mn3-Ca	3.7	3.5	3.3	3.5
Mn4-Ca	4.3	3.8	3.7	3.8

References

Yamashita K, Palmer CM, Burnley T, Murshudov GN. Cryo-EM single-particle structure refinement and map calculation using Servalcat. (2021) *Acta Crystallogr D Struct Biol*. 77(10),1282-1291.

207
